# Mechanistic Investigations Support Liver Safety of Ubrogepant

**DOI:** 10.1093/toxsci/kfaa093

**Published:** 2020-06-24

**Authors:** Brenda Smith, Josh Rowe, Paul B Watkins, Messoud Ashina, Jeffrey L Woodhead, Frank D Sistare, Peter J Goadsby

**Affiliations:** 1 Allergan plc, Irvine, California; 2 Eshelman School of Pharmacy and Institute for Drug Safety Sciences, University of North Carolina at Chapel Hill, Chapel Hill, North Carolina; 3 Department of Neurology, Danish Headache Center, Faculty of Health and Medical Sciences, University of Copenhagen, København, Denmark; 4 DILIsym Services, Durham, North Carolina; 5 Merck & Co., Inc., West Point, Pennsylvania; 6 NIHR-Wellcome Trust King’s Clinical Research Facility, SLaM Biomedical Research Centre, King’s College London, London, UK

**Keywords:** DILIsym, DILI, quantitative systems toxicology, headache, migraine, calcitonin gene-related peptide receptor antagonist

## Abstract

Small-molecule calcitonin gene-related peptide (CGRP) receptor antagonists have demonstrated therapeutic efficacy for the treatment of migraine. However, previously investigated CGRP receptor antagonists, telcagepant and MK-3207, were discontinued during clinical development because of concerns about drug-induced liver injury. A subsequent effort to identify novel CGRP receptor antagonists less likely to cause hepatotoxicity led to the development of ubrogepant. The selection of ubrogepant, following a series of mechanistic studies conducted with MK-3207 and telcagepant, was focused on key structural modifications suggesting that ubrogepant was less prone to forming reactive metabolites than previous compounds. The potential for each drug to cause liver toxicity was subsequently assessed using a quantitative systems toxicology approach (DILIsym) that incorporates quantitative assessments of mitochondrial dysfunction, disruption of bile acid homeostasis, and oxidative stress, along with estimates of dose-dependent drug exposure to and within liver cells. DILIsym successfully modeled liver toxicity for telcagepant and MK-3207 at the dosing regimens used in clinical trials. In contrast, DILIsym predicted no hepatotoxicity during treatment with ubrogepant, even at daily doses up to 1000 mg (10-fold higher than the approved clinical dose of 100 mg). These predictions are consistent with clinical trial experience showing that ubrogepant has lower potential to cause hepatotoxicity than has been observed with telcagepant and MK-3207.

Calcitonin gene-related peptide (CGRP) is a 37-amino acid neuropeptide that plays an important role in migraine pathogenesis. CGRP and its receptor are highly expressed in sensory neurons throughout the peripheral and central trigeminovascular system, mediating vasodilation, and pain signaling in activated nerve fibers (Edvinsson, 2015, 2018; Hargreaves and Olesen, 2019; Russo, 2015). Endogenous CGRP levels are elevated during migraine attacks, and exogenous CGRP has been shown to trigger headaches and delayed migraine-like attacks in people with migraine (Edvinsson, 2015; Goadsby *et al.*, 1990; Lassen *et al.*, 2002).

In recent years, inhibition of CGRP has been identified as a promising therapeutic approach for treating and preventing migraine (Edvinsson, 2018). Several small molecules and monoclonal antibodies targeting CGRP or its receptor have demonstrated clinical efficacy for the acute (Connor *et al.*, 2009; Dodick *et al.*, 2019; Hewitt *et al.*, 2011; Lipton *et al.*, 2019) and preventive (Dodick, 2019; Ho *et al.*, 2014) treatment of migraine. However, development of small-molecule CGRP receptor antagonists was interrupted when clinical safety signals were identified indicating potential liver toxicity for the first-generation compounds, telcagepant and MK-3207 (Hargreaves and Olesen, 2019; Hewitt *et al.*, 2011; Ho *et al.*, 2014).

It was hypothesized that the observed potential liver toxicity of telcagepant and MK-3207 was not an inherent consequence of CGRP receptor inhibition, because α-CGRP knockout mice do not develop liver problems, and because anti-CGRP monoclonal antibodies, such as erenumab, are not associated with liver safety concerns (Dodick *et al.*, 2018; Goadsby *et al.*, 2017; Hargreaves and Olesen, 2019; Walker *et al.*, 2010). Therefore, drug discovery and hepatic safety research efforts continued, with the goal of sufficiently understanding the underlying mechanistic basis for drug-induced liver injury (DILI) to minimize risk for hepatotoxicity while preserving CGRP receptor antagonism (Hargreaves and Olesen, 2019). Based on results of these studies, key characteristics hypothesized to be important for reducing potential hepatotoxicity included greater drug potency, lower dosing while still achieving a similar clinical efficacy, and lower bioactivation potential to form reactive metabolites. As a result of these research efforts, the novel small-molecule CGRP receptor antagonist ubrogepant was developed, and approval for marketing was granted by the U.S. Food and Drug Administration (FDA) for the acute treatment of migraine with or without aura in adults and includes no labeled warnings of liver toxicity potential (Ubrelvy [package insert], 2019).

DILI is one of the most common safety-related reasons for discontinuation or withdrawal of otherwise promising medications (Mosedale and Watkins, 2017; Shoda *et al.*, 2017; Yang *et al.*, 2014). DILI can be a complex and multifactorial process involving the interaction of many different mechanisms. In some cases, DILI is intrinsic (ie, dose-dependent hepatotoxicity that can be elicited in a high proportion of individuals, and may not be observed in animal models); however, DILI can also be idiosyncratic, causing rare cases of serious liver injury in susceptible individuals without a clear dose relationship (Mosedale and Watkins, 2017). Known mechanisms of DILI include, but are not limited to, oxidative stress, development of reactive metabolites, mitochondrial toxicity, altered bile acid homeostasis, and innate and adaptive immune responses (Mosedale and Watkins, 2017). Many pharmaceutical companies have adopted experimental approaches to investigate these mechanisms, but the specific assays are diverse and unevenly applied across the industry (Sistare *et al.*, 2016).

DILIsym is a quantitative system toxicology model that has evolved from a public-private partnership involving scientists from academia, industry, and the FDA (Watkins, 2019). Through creation of simulated patient populations, DILIsym can be used to estimate the frequency and severity of liver toxicity with different medications and dosing regimens, and to compare the hepatic safety between different molecules within the same therapeutic category (Longo *et al.*, 2016; Woodhead *et al.*, 2019). The model can also help identify mechanisms underlying DILI for specific medications (Woodhead *et al.*, 2019), and can be used to interpret discordant liver safety results obtained across animal species (Yang *et al.*, 2014). In addition, DILIsym modeling results can provide insights for interpretation of biomarker data that may help predict liver abnormalities (Church and Watkins, 2018).

Extensive novel platform development and evaluation studies involving over 100 molecules with or without documented clinical DILI were conducted by Merck following the clinical hepatic effects seen with telcagepant and MK-3207. Deemed to have satisfactory performance, the platforms were subsequently applied by Merck scientists to benchmark telcagepant and MK-3207 DILI mechanisms, and subsequently for the DILI derisking, and guided selection of ubrogepant. These initial mechanistic studies concluded that the production of reactive metabolites was a primary causative factor of the clinical DILI observed with telcagepant and MK-3207 and that production of reactive metabolites with ubrogepant in the same test systems was sufficiently reduced to warrant internal approval for nonclinical and clinical development. Those study data and methodologies are published separately, along with the supporting platform development data from over 100 other compounds (Kang *et al.*, forthcoming;Monroe *et al.*, forthcoming;Podtelezhnikov *et al.*, 2020), and the telcagepant, MK-3207, and ubrogepant data are also summarized there. For business reasons, ubrogepant was sold by Merck to Allergan during early clinical development. DILIsym was subsequently engaged by Allergan to generate independent mechanistic assay data used as modeling input for simulations. The DILIsym *in vitro* and *in silico* modeling studies were conducted independently of the Merck mechanistic study data, which were not generated for the purpose of, nor used for, the DILIsym modeling. Here, we present results from nonclinical, *in vitro*, and *in silico* modeling studies that evaluated the hepatic effects of telcagepant, MK-3207, and ubrogepant, including both the Merck mechanistic studies conducted to select and derisk ubrogepant, and the independent DILIsym efforts subsequently conducted.

## MATERIALS AND METHODS

### Preclinical toxicology

Conventional animal toxicology studies in rats and monkeys were performed by Merck & Co, Inc (West Point, Pennsylvania). All animals were cared for and treated in accordance with FDA Good Laboratory Practice for nonclinical laboratory studies. The no-observed-adverse-effect levels, hematological and biochemical parameters (including alanine aminotransferase [ALT] and aspartate aminotransferase [AST]), and pathology parameters were determined for telcagepant, MK-3207, and ubrogepant.

### Human dose determination

Clinically effective doses of telcagepant, MK-3207, and ubrogepant were initially predicted based on preliminary estimates of pharmacokinetic parameters, as well as rhesus monkey capsaicin-induced dermal vasodilation biomarker estimates of *in vivo* pharmacodynamic activity that were corrected for human potency based both on relative differences in antagonism of CGRP-stimulated cyclic adenosine monophosphate responses in human and monkey receptors cloned into human embryonic kidney 293 (HEK293) cells, and species differences in plasma protein binding. Clinically effective doses were later confirmed in initial clinical trials for all 3 compounds.

### Reactive metabolite body burden in human hepatocyte cultures

The “body burden” is a quantitative estimate of reactive metabolite exposure that was determined for each drug based on *in vitro* covalent protein binding of radioactivity in human hepatocytes (in pmol equivalents/mg) multiplied by a clinically effective daily dose (in mg/day). Detailed methods used in the *in vitro* human hepatocyte covalent-binding experiments have been reported by Nakayama *et al.* (2009).

### Reactive metabolite-mediated Nrf2 stabilization and CYP3A4 degradation in HEK cells

A stable HEK293 cell line expressing an inducible CYP3A4 enzyme was used to test the effects of telcagepant, MK-3207, and ubrogepant on nuclear factor erythroid 2-related factor 2 (Nrf2) and CYP3A4 protein levels. Specifically, these assays evaluated the potential of the 3 different CGRP receptor antagonists to form reactive metabolites that promote the stabilization of Nrf2 or covalently bind to CYP3A4, triggering its subsequent time-dependent degradation, as assessed by Western blot. To seek further confirmatory evidence for reactive metabolite formation, a HEK293 cell line expressing doxycycline inducible CYP3A4 P450 enzyme expression construct with a Flag tag epitope fused to the C-terminus was constructed and cloned using the Invitrogen/ThermoFisher Scientific Flp-In T-TEx system and the HEK293 Flp-In host cell line per standard protocol. Because the observed rat liver changes in gene expression are concluded to be driven by reactive metabolites formed from MK-3207 and telcagepant induced largely, but not exclusively, by Nrf2/Keap1 perturbation, Nrf2 stabilization measurable by Western blot using anti-Nrf2 antibody is predicted as an early key molecular event that would be dependent on both presence of parent drug and the metabolic machinery (presumed to be CYP3A4) being present to bioactivate parent to a chemically reactive intermediate. Alternatively, this simplified single CYP test model might not allow for Nrf2 stabilization if sufficient quantities of the chemically reactive metabolite cannot be generated by CYP3A4, or cannot escape the catalytic site of CYP3A4, which would appear on Western blot using anti-Flag antibody as time-dependent CYP3A4 degradation. These data have been presented previously (Monroe *et al.*, 2018, forthcoming).

### Gene expression of rat liver response to bioactivation

Global gene expression profiling was performed on rat livers collected following oral administration of high doses of the different CGRP receptor antagonists (7 days with 750 mg/kg MK-3207 or ubrogepant, and 4 days with a maximally tolerated dose of 300 or 400 mg/kg telcagepant). A prediction of a clinical daily dose burden associated with reactive metabolite-mediated DILI potential was derived from a focused and integrated quantitative rat liver molecular gene expression signature optimized predominantly for Nrf2 electrophilic stress and Nrf1 proteasomal stress pathways to describe a bioactivation liver response assay (BA-LRA) result. Clinical risk is derived based on an evaluation of results from over 100 test compounds used to optimize the calculation that uses the strength of the measured gene expression signature together with the clinical daily dose liver burden. The clinical dose is identified that projects DILI risk to exceed the optimized threshold calculation for each BA-LRA result. These data have been presented previously (Monroe *et al.*, 2018, forthcoming; Podtelezhnikov *et al.*, 2018, 2020).

### Gene expression of *in vitro* hepatocyte response to bioactivation using rat HEPATOPAC

Targeted gene expression to assess the same bioactivation response mechanisms (Nrf2 oxidative stress and Nrf1 proteasomal stress pathways) was evaluated for telcagepant and MK-3207 in a rat hepatocyte micropatterned coculture model (HEPATOPAC; BioIVT, Medford, Massachusetts) and have been presented previously (Kang *et al.*, 2018, forthcoming); ubrogepant was not evaluated in these studies.

### *In vitro* bile acid transporter effects in human HEPATOPAC model

The human HEPATOPAC model was also used to measure the effects of test agents on transport of taurocholic acid as an indicator of altered bile salt export pump (BSEP) function (Li *et al.*, 2017). Safe dose perspective is gained from comparing *in vitro* concentration response results to calculated liver inlet *C*_max_ (maximum plasma concentration).

### Mitochondrial function *in vitro* in rat and human HEPATOPAC model

Effects of test agents on mitochondrial function were assessed from media collected in the same HEPATOPAC studies by monitoring urea synthesis rates (Khetani *et al.*, 2013).

### DILIsym modeling

The DILIsym (DILIsym Services, Inc, Research Triangle Park, North Carolina) mechanistic mathematical model was used to assess the predicted risk of hepatotoxicity of ubrogepant compared with telcagepant and MK-3207. DILIsym can translate *in vitro* mechanistic assay data into predictions of hepatotoxicity based on liver exposure estimates derived from physiologically based pharmacokinetic modeling, proposed dosing regimens, nonclinical and clinical metabolism data, and quantitative data collected in *in vitro* experimental systems (Mosedale and Watkins, 2017; Woodhead *et al.*, 2017). Commercial and academic versions of the DILIsym software are available and can be obtained through www.simulations-plus.com/software/dilisym. DILIsym v7A was used to perform the simulations for ubrogepant and MK-3207; DILIsym v5A was used to perform the simulations for telcagepant. Differences between versions were not mechanistically relevant to the data reported and did not impact the results.

The *in vitro* data used in the DILIsym models for ubrogepant, telcagepant, and MK-3207 were generated from experiments measuring the potential for each drug to cause hepatotoxicity via bile acid transporter inhibition, mitochondrial dysfunction, and oxidative stress (Supplementary Material). *In vitro* assay results were incorporated in the DILIsym software, along with estimates of dosing-dependent drug exposure inside and outside hepatocytes. The effects of each compound were simulated at clinically relevant and supratherapeutic doses, and results were compared across compounds and against previous clinical trial results. The parameters within DILIsym have been varied to reflect genetic and nongenetic factors that underlie variation in individual susceptibility (ie, the model generates simulated patient populations). The primary outputs from DILIsym are serum ALT, which reflects death of hepatocytes and release of this biomarker into blood, and bilirubin levels, which rise based on the loss of global liver function predicted from the reduction in viable hepatocytes. The results obtained in the simulated populations are expressed in terms of the percentage of individuals with changes in serum ALT or bilirubin exceeding arbitrary thresholds. The results are also displayed in graphic form according to the FDA standard diagram format known as evaluation of drug-induced serious hepatotoxicity (eDISH) (Watkins *et al.*, 2011). An eDISH diagram plots peak serum ALT versus total bilirubin levels observed in each clinical trial subject on log scales. eDISH plots divide results into 4 quadrants: (1) serum ALT ≤ 3× the upper limit of normal (ULN) and total bilirubin ≤ 2× ULN; (2) isolated hyperbilirubinemia, defined as total bilirubin > 2× ULN and serum ALT ≤ 3× ULN; (3) serum ALT > 3× ULN and serum bilirubin ≤ 2× ULN, which indicates hepatocellular injury without global liver dysfunction (also termed Temple’s Corollary quadrant); and (4) serum ALT > 3× ULN and total bilirubin > 2× ULN, which indicates both hepatocellular injury and global liver dysfunction qualifying as Hy’s Law cases (Senior, 2014; Watkins *et al.*, 2011). Drug treatment that results in data points in the Temple’s Corollary quadrant is associated with increased risk of DILI, and drug treatment that results in data points in the Hy’s Law quadrant is associated with an increased risk of liver failure (Regev and Bjornsson, 2014; Watkins *et al.*, 2011).

The dominant mechanisms accounting for liver toxicity predicted by DILIsym are determined by sequentially turning off each of the 3 mechanisms and assessing the effect on the incidence of toxicity in the simulated patient populations. A decrease in the simulated incidence of toxicity in the absence of a mechanism indicates that the mechanism is predicted to be involved in the observed toxicity.

## RESULTS

Results from conventional nonclinical animal toxicology studies, each conducted in 2 or more species, did not reveal liver safety liabilities for telcagepant, MK-3207, or ubrogepant (Table 1). Mechanistic experiments conducted by Merck to assess the mitochondrial toxicity potential (Xu *et al.*, 2019) of telcagepant and MK-3207 in a HepG2 cell glucose/galactose shift model did not raise mitochondrial safety concerns. Experiments conducted in HEPATOPAC with telcagepant and MK-3207 similarly did not raise concern for mitochondrial-based safety liabilities, based on absence of perturbation of urea synthesis (Table 1). Experiments designed to more generally assess BSEP transport inhibition and perturbation of bile acid homeostasis, both *in vitro* (Table 1) and *in vivo* in rats (Li *et al.*, 2019), were negative or were interpreted as unlikely to be clinically relevant for telcagepant. For MK-3207, *in vitro* studies of bile acid transport inhibition conducted in vesicles and human HEPATOPAC raised minimal concern. For MK-3207 at the highest testable concentration (due to solubility limitations) of 4.4 times the calculated unbound liver inlet concentration, no significant impact was observed on biliary excretion (Table 1) in human HEPATOPAC. Parameters reflecting the *in vitro* abilities of the 3 molecules to generate reactive metabolites and electrophilic stress were consistent in demonstrating DILI risk for MK-3207 and telcagepant, and are summarized in Table 1 and further described elsewhere (Kang *et al.*, forthcoming;  Monroe *et al.*, forthcoming). The calculated reactive metabolite body burden for ubrogepant (4600) was lower than for telcagepant (14 560) or MK-3207 (14 720), indicating that ubrogepant has a lower potential to form reactive metabolites at dosing likely to achieve therapeutic results. Consistent with these findings, results from rat liver gene expression studies indicated that telcagepant and MK-3207 upregulated pathways associated with electrophilic and proteasomal stress, whereas ubrogepant did not. For MK-3207, doses > 100 mg daily were predicted to cause DILI based on Nrf1 and Nrf2 *in vivo* quantitative rat liver gene expression. In addition, results from the HEK293/CYP3A4 assay showed that MK-3207 formed reactive metabolites that covalently bind to CYP3A4 and result in its degradation, whereas ubrogepant and telcagepant did not. Transcriptional responses consistent with reactive metabolites and/or electrophilic stress were also observed for telcagepant and MK-3207 (Kang *et al.*, 2018, forthcoming) using the HEPATOPAC micropatterned hepatocyte coculture system (ubrogepant was not tested in these experiments). Follow-up studies using MK-3207 with radiolabels strategically targeted to 2 positions on the molecule based on metabolism ID study data from the rat BA-LRA study confirmed the presence of 2 chemically reactive sites on the molecule (Monroe *et al.*, 2018, forthcoming, Table 1). 

**Table 1.** Summary Results of Pivotal *In Vivo* Toxicology, Clinical Study, *In Vivo* Rat BA-LRA, and *In Vitro* Rat HEPATOPAC Studies Conducted for Telcagepant, MK-3207, and Ubrogepant

Parameter	Telcagepant^a^	MK-3207^b^	Ubrogepant^c^
Structure^d^	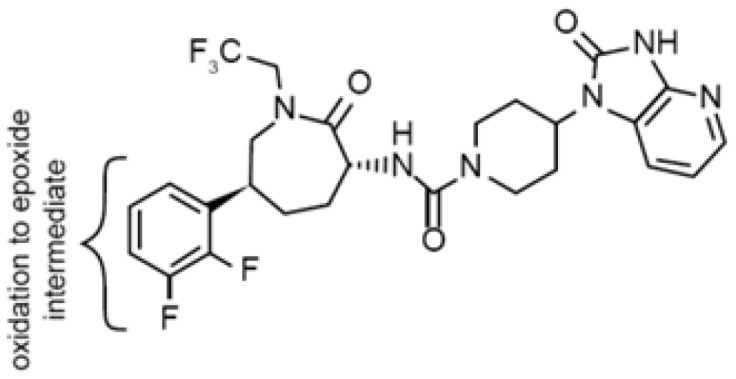	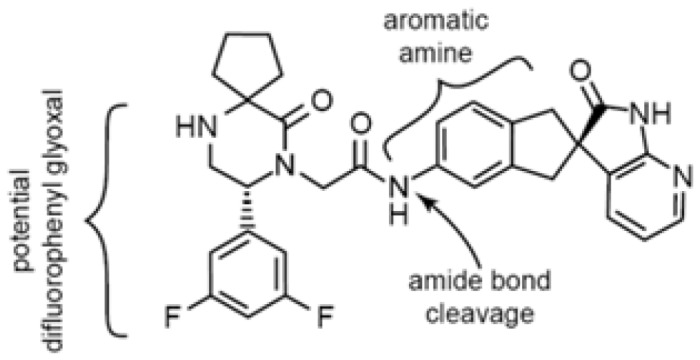	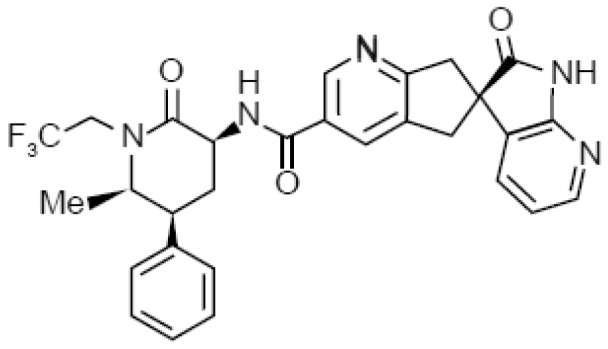
Potency IC_50_^e^	2.2 nM	0.12 nM	0.08 nM
Pivotal conventional nonclinical toxicology study liver findings	3M rat: < 3× ALT/AST with no liver histopathology at 15× exposure margin 6M rat: no liver safety signal at 7× margin 9M NHP: no liver safety signal at 7× margin 6M mouse: < 2× ALT/AST with no liver histopathology at 14× margin	6M rat: no liver safety signal at 25× exposure margin 9M NHP: no liver safety signal at 4× margin 6M mouse: no liver safety signal at 12× margin 1M dog: slight periportal vacuolation with < 4× ALT/AST associated with excessive body weight loss at 17× margin	6M rat: < 2× ALT with no liver histopathology at 70× exposure margin 9M NHP: no liver safety signal at 163× margin 3M mouse: no liver safety signal at 80× margin
Pivotal clinical study findings	ALT > 3-fold ULN significantly increased after 2 weeks in 3.2% at 280 mg BID, with 2 cases of symptomatic hepatitis with ALT rises ≥ 10-fold. ALT rises generally occurred while on drug and resolved rapidly on discontinuation. Discontinued in phase 3.	ALT > 3-fold ULN after 2 weeks in 1% at daily doses of < 100 mg and in 42% (5/12) > 500 mg. Among these 5 patients were 3 with > 20-fold ALT rises, 1 symptomatic with Hy’s law. ALT rises generally were delayed in onset (up to 2 and 3 months) and slow to resolve. Discontinued in phase 2.	ALT/AST ≥ 3-fold ULN in 5 (1.9%) placebo and 2 (0.8%) ubrogepant participants after intermittent, high-frequency dosing (100 mg QD for 2 days, then placebo for 2 days, alternating). Both ubrogepant cases were asymptomatic, showed no concurrent bilirubin elevations, and resolved despite continued dosing.
*In vivo* rat gene expression BA-LRA of electrophilic stress	BA-LRA score 0.30 at 400 mg/kg/day × 4 days with evidence of transcriptional suppression, so maximum liver safe daily dose expectation was <600 mg	BA-LRA score 0.34 at 600 mg/kg/day × 4 days predicting maximum liver safe daily dose boundary of 300 mg	No BA-LRA response
*In vitro* rat HEPATOPAC study of: (a) gene expression BA-LRA of electrophilic stress, (b) mitochondrial urea synthesis, and (c) bile acid excretion	BA-LRA score > 0.2 at 50 μM No effect on urea at 50 µM (mitochondrial function) No effect on bile acid excretion at 10.7× estimated unbound liver *C*_max_	BA-LRA score > 0.2 at 50 μM No effect on urea at 50 µM (mitochondrial function) Slight effect on bile acid excretion at 4.4× estimated unbound liver *C*_max_	Not done
Projected body burden from covalent-binding studies^f^	14 560 (560 mg × 26)	14 720 (80 mg × 184)	4600 (200 mg × 23)

a Phase 3 clinical study exposures based on mean of 70 μM/h at dose of 280 mg BID (2 h apart).

b Phase 2 clinical study exposures based on mean of 60 μM/h at dose of 900 mg daily; original projected daily dose was 80 mg.

c Phase 3 clinical study exposures based on 2 μM/h at dose of 100 mg QD.

d Text corresponds to potential reactive metabolite pathways.

e CGRP-stimulated cAMP response in HEK293 cells.

f Body burden = covalent protein binding × dose. Doses: telcagepant 280 mg with potential redosing (560 mg); MK-3207 80 mg QD; ubrogepant 100 mg with potential redosing (200 mg).

Abbreviations: ALT, alanine aminotransferase; AST, aspartate aminotransferase; BA-LRA, bioactivation-liver response assay; BID, twice daily; cAMP, cyclic adenosine monophosphate; CGRP, calcitonin gene-related peptide; *C*_max_, maximum plasma concentration; HEK293, human embryonic kidney 293; IC_50_, half-maximal inhibitory concentration; M, male; NHP, nonhuman primate; QD, once daily; ULN, upper limit of normal.

**Figure 1.**
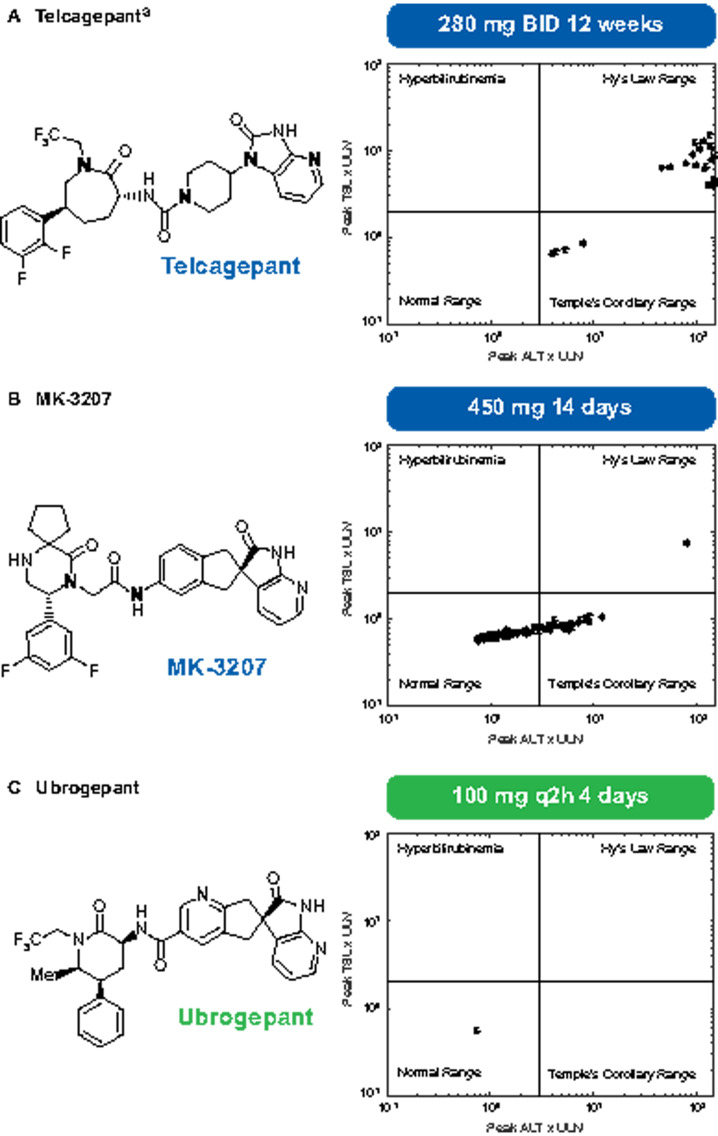
DILIsym generated eDISH plots of telcagepant (A), MK-3207 (B), and ubrogepant (C) treatment in simulated patient populations. ^a^For telcagepant, eDISH simulations were run for responders; nonresponders (ALT <3× ULN) to telcagepant at 280 mg BID over 12 weeks are not shown. Abbreviations: ALT, alanine aminotransferase; eDISH, evaluation of drug-induced serious hepatotoxicity; q2h, 2 doses of 100 mg separated by 2 h (200 mg/day); TBL, total bilirubin; ULN, upper limit of normal.

DILIsym modeling, which incorporates quantitative assessments of oxidative stress, mitochondrial dysfunction, and disruption of bile salt homeostasis, predicted telcagepant and MK-3207 would be hepatotoxic at pharmacologically relevant doses, which was confirmed with observations from clinical trials of these drugs (Figs. 1A and 1B). Specifically, telcagepant was predicted to be hepatotoxic at clinical doses of ≥ 175 mg BID. A total of 36 simulated individuals treated with telcagepant 280 mg BID over 12 weeks were predicted to develop ALT > 3× ULN, with many simulated individuals meeting the criteria for Hy’s Law cases (Table 2). This hepatotoxicity was predicted to be driven predominantly by bile acid accumulation, with lesser contribution to hepatotoxicity reflecting mitochondrial electron transport chain inhibition. Oxidative stress was not predicted to contribute to telcagepant’s hepatotoxicity in DILIsym. 

**Table 2.** DILIsym Predictions for Telcagepant, MK-3207, and Ubrogepant in a Simulated Patient Population of Healthy Volunteers

Compound	Dosing Protocol	Simulated ALT > 3× ULN^a^	Clinical ALT > 3× ULN
Telcagepant^b^	280 mg BID 12 weeks	12.6% (36/285)	3.2% (8/253) (Ho *et al.*, 2014)
	140 mg BID 12 weeks	0% (0/285)	1.9% (5/258) (Ho *et al.*, 2014)
MK-3207^c^	200 mg q2h, 2 daily doses (400 mg daily dose), for 14 days	3.5% (10/285)	42% (5/12) among individuals dosed for more than 1 week; most responding were given 600–900 mg per day
	300 mg q2h, 2 daily doses (600 mg daily dose), for 14 days	7% (20/285)	
	450 mg q2h, 2 daily doses (900 mg daily dose), for 14 days	10.2% (29/285)	
Ubrogepant	100 mg q2h, 4 days	0% (0/285)	N/A
	1000 mg q2h, 4 days	0% (0/285)	N/A
	100 mg QD, 8 days	0% (0/285)	N/A
	1000 mg QD, 8 days	0% (0/285)	N/A
	50 mg QD, 2 days on, 2 days off for 56 days, 28 total doses	0% (0/285)	N/A
	100 mg QD, 2 days on, 2 days off for 56 days, 28 total doses	0% (0/285)	0.8% 2/256 (Goadsby *et al.*, 2019)
	1000 mg QD, 2 days on, 2 days off for 56 days, 28 total doses	0% (0/285)	N/A
	50 mg q2h, 28 straight days, 56 total doses	0% (0/285)	N/A

a ULN in DILIsym is 40 U/l.

b The mechanisms involved in the predicted liver injury for telcagepant were mainly inhibition of bile salt transport, with a lesser contribution of mitochondrial electron transport inhibition and no contribution of oxidative stress.

c The mechanisms involved in the predicted liver injury for MK-3207 were competitive bile salt export pump inhibition and inhibition of mitochondrial electron transport, with oxidative stress being a minor contributor.

Abbreviations: ALT, alanine aminotransferase; ULN, upper limit of normal.

DILIsym modeling results for MK-3207 at doses of 200, 300, or 450 mg given 2 h apart twice per day for 14 days was predicted to cause ALT elevations, which is consistent with clinical observations. Using simulated treatment with the highest dose of MK-3207 (450 mg over 14 days), DILIsym predicted ALT elevations > 3× ULN in 10.2% (29/285) of a simulated patient population of healthy volunteers. When the MK-3207 dose was decreased to 200 mg, 3.5% (10/285) of simulated individuals were predicted to experience ALT levels > 3× ULN. At the 450-mg dose level, the DILIsym model predicted 1 individual with bilirubin elevations > 2× ULN concomitantly with ALT > 3× ULN (ie, a Hy’s Law case) (Table 2). MK-3207 hepatotoxicity was also predicted to be mainly driven by bile acid accumulation and mitochondrial electron transport chain inhibition, and oxidative stress was predicted to be a minor contributor to hepatotoxicity.

In contrast to the DILIsym results obtained for telcagepant and MK-3207, DILIsym predicted ubrogepant would be safe for the liver in all simulated individuals (Figure 1C), with a large margin of safety. No ALT elevations were predicted at ubrogepant doses of up to 1000 mg, which is 10-fold higher than the proposed clinical dose of 100 mg (Table 2).

DILIsym also predicted total liver safety of ubrogepant 200 mg daily (two 100 mg doses separated by 2 h) for 4 days and following supratherapeutic high-frequency intermittent dosing (2 days of 1000 mg/day followed by 2 days off, for a total of 56 days [28 total doses]).

## DISCUSSION

The small-molecule CGRP receptor antagonists telcagepant and MK-3207 have demonstrated clinical efficacy for the treatment of migraine; however, their development was terminated after hepatotoxicity was observed with repeated use (Hargreaves and Olesen, 2019). Thus, a key component in the development of ubrogepant has been rigorous evaluation of DILI risk.

The integrated mechanistic data set available at the time of ubrogepant candidate selection indicated DILI risk for MK-3207 and telcagepant was high for a bioactivation-mediated mechanism. The development of ubrogepant was, therefore, predicated on the hypothesis that the liver toxicity of telcagepant and MK-3207 was attributable, at least partly, to reactive metabolites. Results from *in vitro* studies and covalent-binding experiments confirmed ubrogepant has higher target engagement potency than telcagepant, allowing for administration of lower doses for therapeutic efficacy and thereby reducing the potential exposure to reactive metabolites (ie, body burden). Furthermore, findings from *in vitro* and *in vivo* rat liver gene expression studies indicated that, unlike telcagepant and MK-3207, ubrogepant does not trigger upregulation of pathways associated with electrophilic and proteasomal stress, and HEK293/CYP3A4 assay results indicated ubrogepant does not form reactive metabolites that covalently bind to CYP3A4, as was seen with MK-3207. It is important to understand that the *in vivo* and *in vitro* gene expression data, radiolabel studies, and HEK293 studies referred to above, are not traditional, routine regulatory study data. Instead, there is much debate and lack of alignment among regulatory and industry toxicologists over the practical value and utility of such mechanistic assay data. In addition, these investigations were not launched until after MK-3207 and telcagepant presented with clinical DILI and their development had been discontinued, so these comparative data were only available for regulatory reviewers at the time of ubrogepant’s nonclinical development.

DILIsym results utilizing data from different *in vitro* experimental models and estimates of dose-dependent liver exposure predicted liver safety liabilities of telcagepant and MK-3207 and no significant liver toxicity with repeated administration of ubrogepant at daily doses much higher than expected clinically. Together, the results of these nonclinical, *in vitro*, and *in silico* modeling studies support the relatively benign liver safety profile of ubrogepant that has been established in clinical trials.

DILIsym modeling addressed 3 main causes of DILI: mitochondrial dysfunction, oxidative stress, and bile acid transporter disruption (Shoda *et al.*, 2017). The mechanistic candidate selection studies and the DILIsym modeling results showed reduced potential for activation of these mechanisms of liver toxicity for ubrogepant compared with telcagepant and MK-3207. As the causes of DILI are often multifactorial (Ghabril *et al.*, 2010), it is noteworthy that ubrogepant displayed a lower potential for hepatotoxicity across all mechanisms. In general, these findings strongly support the improved hepatic safety profile of ubrogepant compared with telcagepant and MK-3207.

Overall, DILIsym results were well correlated with clinical hepatic safety data, supporting the validity of DILIsym modeling for this class of compounds. For telcagepant, DILIsym predicted hepatotoxicity at clinical doses ≥ 175 mg BID, consistent with results from a phase 2 study for the acute treatment for migraine in which 13 of 638 people experienced ALT elevations ≥ 3× ULN, including 1.9% (5/258) of those treated with telcagepant 140 mg BID and 3.2% (8/253) of those treated with telcagepant 280 mg BID, compared with 0 of 127 people randomized to placebo (Ho *et al.*, 2014). DILIsym results also correctly predicted ALT elevations with MK-3207; however, the model predicted elevations at a lower frequency than observed in MK-3207 clinical trials. These differences possibly may be explained by an immune-mediated component not accounted for in DILIsym modeling or by a toxic stable metabolite that was not investigated.

The lack of hepatotoxicity predicted by DILIsym for ubrogepant in this study was confirmed with data from 2 pivotal, randomized, controlled, phase 3 trials (ACHIEVE I and II) (Dodick *et al.*, 2019; Lipton *et al.*, 2019) and a long-term extension trial (Ailani *et al.*, 2019) in people with migraine, and a phase 1 hepatic safety trial in healthy adults (Goadsby *et al.*, 2019). In the ACHIEVE trials, ubrogepant was safe and well tolerated for the acute treatment of migraine (Dodick *et al.*, 2019; Lipton *et al.*, 2019). Across all treatment arms including placebo, 6 cases of ALT or AST elevation ≥ 3× ULN were identified in ACHIEVE I, and 4 in ACHIEVE II. These events were evaluated and adjudicated by a panel of liver experts blinded to treatment allocation, and no cases (ubrogepant or placebo) were adjudicated as “probably related” to treatment. In a 52-week extension trial, 1230 people who experienced 22 454 migraine attacks received 31 968 doses of ubrogepant (Ailani *et al.*, 2019). There were no signals for DILI or any hepatic safety concerns. In this long-term extension trial, 20 cases of ALT or AST ≥ 3× ULN were reported and adjudicated. One case was judged “probably related” to treatment, but with confounding factors present. All cases were asymptomatic, with no concurrent bilirubin elevations and all ALT and AST elevations resolved in those who continued ubrogepant treatment (Ailani *et al.*, 2019). The most rigorous test of the liver safety of ubrogepant was a randomized, double-blind, placebo-controlled, 8-week dedicated hepatic safety trial in which 516 healthy adults received placebo (*n* = 260) or intermittent, high-frequency dosing with ubrogepant (*n* = 256; 2 consecutive days of treatment with ubrogepant 100 mg alternating with 2 days of placebo) (Goadsby *et al.*, 2019). Ubrogepant was well tolerated, with no signal for DILI or hepatic safety concerns. Over the 8 weeks of treatment, 7 cases of ALT or AST ≥ 3× ULN were observed (5 in the placebo group, 2 in the ubrogepant group), with 4 adjudicated “unlikely related,” 2 “possibly related,” and 1 “probably related” to treatment by 2 hepatologists and “possibly related” by a third hepatologist. All cases were asymptomatic, no cases had concurrent bilirubin elevation, and none met international criteria for DILI (Goadsby *et al.*, 2019). To our knowledge, this study is the first published example of DILIsym predicting liver safety of a dosing regimen before the clinical trial was conducted.

Overall, the Merck preclinical derisking experiments and DILIsym model output results agree with clinical data for telcagepant, MK-3207, and ubrogepant. Nevertheless, there are several caveats, including an incomplete understanding of the mechanisms causing DILI and a gap in the availability of confirmatory translational biomarkers that could provide mechanistic insight into DILI, and controversy over the relative importance of each known mechanism. Reliable models are unavailable for predicting a drug’s effect on the innate and adaptive immune system and reflect such a clinical phenotype of reactive metabolite formation. For the *in vitro* and *in vivo* models that are available, there is much variability in how the studies are conducted, and the data analyzed and interpreted. The potential effects of stable toxic metabolites are not represented in many of the model systems, though this is improving as more phenotypically stable liver models, such as HEPATOPAC, are developed and refined. Furthermore, there are alternative, known mechanisms contributing to DILI, and additional mechanisms that contribute to DILI likely will be identified. Results of this study should be interpreted with the understanding that mechanistic *in vitro* DILI derisking assays and DILIsym modeling can only incorporate known hepatotoxicity mechanisms, and details of the *in vitro* assay conduct and underlying data input will impact model outcome. As with any model, the validity of the predicted results is a function of the strength and accuracy of the chosen input variables. Whereas DILIsym model output results in this study agree with existing clinical data for telcagepant, MK-3207, and ubrogepant, the inputs for these models were limited to data from *in vitro* experiments. Significant differences in the experimental protocols and thresholds used between Merck and DILIsym scientists for assessing *in vitro* mitochondrial and BSEP inhibition potential may also help account for the difference between the initial mechanistic assay conclusions from Merck for MK-3207 and telcagepant and the DILIsym mechanistic predictions.

Furthermore, DILIsym predictions of the severity of liver injury should be interpreted with caution. The severity of hepatotoxicity may be overestimated with DILIsym because clinical stopping rules are not employed. In clinical practice, treatment may be discontinued at the first sign of ALT elevation; however, DILIsym models are based on continued dosing. In addition, when these analyses were conducted, DILIsym did not fully represent several adaptive mechanisms that could mitigate toxic responses to pharmacotherapy, such as mitochondrial biogenesis in response to inhibition of mitochondrial function (Woodhead *et al.*, 2019). Finally, whereas there is sound scientific rationale for the concept that lower bioactivation potential and increased potency to reduce total body burden will reduce total liver exposure to reactive intermediates, it is important to acknowledge that the precise mechanisms by which reactive metabolites trigger hepatocellular injury causing aminotransferase elevations with telcagepant and MK-3207 are unclear. Attempting to predict the impact that structural or pharmacokinetic differences between CGRP receptor antagonists will have on liver safety is challenging, but also potentially rewarding if considered before candidate selection, as this could reduce ultimate safety-related attrition. Any novel medication should undergo comprehensive testing to evaluate its potential effect on hepatic function. Using the data generated from long-term clinical trials and hepatic safety assessments noted herein, ubrogepant was approved by the FDA without label precautions for liver safety (Ubrelvy [package insert], 2019).

In summary, ubrogepant is a novel, small-molecule CGRP receptor antagonist that is chemically distinct from previous CGRP receptor antagonists. Ubrogepant was developed as a result of intensive mechanistic investigations with the goal of selecting a compound with reduced DILI potential, with a focus on lower potential to form reactive metabolites. In this study, DILIsym modeling reproduced the hepatotoxicity of previous CGRP receptor antagonists but predicted ubrogepant to be safe, even at doses greatly exceeding those that are efficacious. These data further support the positive liver safety profile of ubrogepant demonstrated in clinical trials.

## SUPPLEMENTARY DATA

Supplementary data are available at *Toxicological Sciences* online.

## Supplementary Material

kfaa093_supplementary-data
