# Supplementary material for: Mechanistic Investigations Support Liver Safety of Ubrogepant
Source: Toxicol Sci. 2020 Jun 24;177(1):84–93. doi: 10.1093/toxsci/kfaa093 (PMC8312697; doi:10.1093/toxsci/kfaa093)
Supplement: kfaa093_supplementary-data [file kfaa093_supplementary-data.docx]

# **Supplementary Materials**

Mechanistic Investigations Support Liver Safety of Ubrogepant

Brenda Smith, PhD, DABT*; Josh Rowe, BSc*; Paul B. Watkins, MD; Messoud Ashina, MD, PhD, DMSc; Jeffrey L. Woodhead, PhD; Frank D. Sistare, PhD; Peter J. Goadsby, MD, PhD

# **In Vitro Assay Descriptions**

### Bile acid transporter inhibition by telcagepant

To assess the functional impact of telcagepant on the human ATP-binding cassette (ABC) efflux transporters BSEP, MRP3, and MRP4, *in vitro* vesicular transport inhibition assays were conducted by Solvo Biotechnology (Szeged, Hungary). The ability of telcagepant to inhibit the BSEP-, MRP3-, or MRP4-mediated transport (and accumulation) of a probe substrate was tested at concentrations ranging from 0.03 – 20 μM, where the latter defined the upper limit of compound solubility. To assess the functional impact of telcagepant on the human SLC (uptake) transporter NTCP, *in vitro* experiments were performed using CHO cells stably expressing NTCP. The ability of telcagepant to inhibit NTCP‑mediated uptake (and accumulation) of taurocholate was tested at concentrations ranging from 0.03 – 25 μM.

For the simulation of bile acid transporter inhibition by telcagepant in DILIsym®, parameter values must be selected for BSEP canalicular transport, basolateral transport (encompassing both MRP3 and MRP4), and NTCP transport. The parameter values for each include the inhibition constant, defined as the IC_50_ or K_i_ value, and the inhibition type, defined as competitive, non-competitive, or mixed inhibition. For a given inhibition constant, the inhibition type has been shown to influence bile acid accumulation.^1,2^ To illustrate, for a given inhibition constant, non-competitive BSEP inhibition by a compound will lead to greater bile acid accumulation than competitive BSEP inhibition. This is because as intracellular bile acid concentrations rise, they can successfully compete for BSEP binding with a competitive inhibitor, whereas non-competitive inhibition cannot be overcome by the rise in intracellular bile acids. The inhibition type can be determined from experimental K_i_, but not IC_50_, studies.

In the current project, additional assays were conducted to determine K_i_ and inhibition type of telcagepant for BSEP-mediated bile acid transport. These assays were conducted by Solvo Biotechnology and overseen by DSSI. Briefly, BSEP inhibition studies were performed with multiple substrate concentrations (0.24 – 19.69 µM taurocholate) and multiple inhibitor concentrations (2.5 – 20 µM telcagepant). K_i_ and inhibition type of telcagepant were determined by fitting a mixed inhibition model to the untransformed data by nonlinear regression analysis. It was assumed that inhibition type of telcagepant for other transporters (i.e., MRP3, MRP4, and NTCP) were the same as inhibition type of telcagepant for BSEP.

### Mitochondria toxicity due to telcagepant

To assess the potential mitochondrial liabilities of telcagepant, cellular respiration assays using a Seahorse XFe96 Flux Analyzer were conducted by Cyprotex (Macclesfield, UK). HepG2 cells were incubated with telcagepant, at concentrations ranging from 0.04 – 150 μM, for 1 or 24 hours, and tested for an effect on cellular respiration as measured by the oxygen consumption rate (OCR). Identical parallel cultures were established to test for an effect on cytotoxicity via cellular ATP content. Three independent studies were conducted in triplicate. Importantly, the 1-hour culture was conducted in the absence of serum (i.e., no serum protein binding) as is standard for the Seahorse Analyzer assay conditions. However, the 24-hour culture was too long for robust cell culture in the absence of serum. Serum was present up to 1 hour before the assay, when cell culture media was exchanged for the standard assay media. Three independent studies were conducted in duplicate.

The parameter values for telcagepant were determined by reproducing the cellular respiration data in MITOsym®, a mechanistic model of mitochondrial function in the in vitro setting. MITOsym® parameters were then translated to DILIsym® parameters using a scaling factor calculated using classic exemplar compounds that perturb different pathways of mitochondrial respiration (e.g., rotenone for ETC inhibition, FCCP for uncoupling).

The concentration of test compound at the site of action (i.e., hepatocytes) has been identified as highly influential in the selection of parameter values. To assess intracellular concentration of telcagepant, identical HepG2 cultures were established in parallel and cell lysate concentrations of telcagepant were measured by LC/MS/MS analysis. Intracellular concentrations of telcagepant were calculated by correcting for cell lysate volume (100 µL) and volume of HepG2 cells in each well, which was calculated by multiplying cell count per well by cellular volume per cell (2.85 pl/cell). Non-specific binding to the culture plates was corrected by subtracting telcagepant concentrations in the no-cell plates from those in cell lysates. One independent study in duplicate was conducted to assess intracellular concentrations of telcagepant.

### Oxidative stress due to telcagepant

To assess the potential for telcagepant to induce oxidative stress, assays to measure formation of reactive oxygen species (ROS) were conducted by Cyprotex. HepG2 cells were cultured in triplicate with telcagepant for 1 or 24 hours, at concentrations ranging from 0.004 – 150 μM, and ROS formation was assayed by DHE fluorescence assay. To include the possible contribution of a telcagepant metabolite to the induction of oxidative stress, a parallel study was conducted in cryopreserved primary human hepatocytes. Three independent studies in triplicate were conducted on HepG2 cells, and two independent studies in triplicate were conducted on primary human hepatocytes.

Parameter values for telcagepant-mediated induction of oxidative stress were identified by simulating the experimental data in DILIsym® using a DILIsym® dosing scheme meant to represent *in vitro* conditions.

The concentration of test compound at the site of action (i.e., hepatocytes) has been identified as highly influential in the selection of parameter values. To assess intracellular concentration of telcagepant, identical HepG2 or primary human hepatocyte cultures were established in parallel and cell lysate concentrations of telcagepant were measured by LC/MS/MS analysis. Intracellular concentrations of telcagepant were calculated by correcting for cell lysate volume (100 µL) and volume of HepG2 cells or hepatocytes in each well, which was calculated by multiplying cell count per well by cellular volume per cell (2.85 pl/cell for HepG2 and 6.46 pl/cell for primary human hepatocytes). Non-specific binding to the culture plates was corrected by subtracting telcagepant concentrations in the no-cell plates from those in cell lysates. One independent study in triplicate was conducted to assess intracellular concentrations of telcagepant for HepG2 and primary human hepatocytes, respectively.

### Bile acid transporter inhibition by ubrogepant

To assess the functional impact of ubrogepant on the human ABC efflux transporters BSEP, MRP3, and MRP4, *in vitro* vesicular transport inhibition assays were conducted by Solvo Biotechnology (Szeged, Hungary). The ability of ubrogepant to inhibit the BSEP-mediated transport (and accumulation) of a probe substrate was tested at concentrations ranging from 0.04 – 30 μM, where the latter defined the upper limit of compound solubility in the BSEP assay buffer. For the MRP3 and MRP4 vesicle-based transporter assessments, ubrogepant concentrations of 0.1 to 70 μM were used. To assess the functional impact of ubrogepant on the human SLC (uptake) transporter NTCP, *in vitro* experiments were performed using HEK293 cells stably expressing NTCP. The ability of ubrogepant to inhibit NTCP‑mediated uptake (and accumulation) of taurocholate was tested at concentrations ranging from 0.04 – 30 μM.

In the current project, additional assays were conducted to determine the K_i_ value and inhibition type for ubrogepant for BSEP-mediated bile acid transport. These assays were conducted by Solvo Biotechnology and overseen by DSSI. Briefly, BSEP inhibition studies were performed with multiple substrate concentrations (0.39 – 50 µM taurocholate) and multiple inhibitor concentrations (0.5 – 30 µM ubrogepant). K_i_ and inhibition type for ubrogepant were determined by fitting a mixed inhibition model to the untransformed data by nonlinear regression analysis. It was assumed that inhibition type for ubrogepant for other transporters (i.e., MRP3 and MRP4) was mixed with α= 5; this is a typical value for bile acid transporter inhibitors in the experience of the DSS team.

### Mitochondria toxicity due to ubrogepant

To assess the potential mitochondrial liabilities of ubrogepant, cellular respiration assays using a Seahorse XFe96 Flux Analyzer were conducted by Cyprotex (Macclesfield, UK). HepG2 cells were incubated with ubrogepant at concentrations ranging from 0.1 – 125 μM for 1 or 24 hours and tested for an effect on cellular respiration as measured by the oxygen consumption rate (OCR). Three independent studies were conducted in duplicate. Importantly, the 1-hour culture was conducted in the absence of serum (i.e., no serum protein binding) as is standard for the Seahorse Analyzer assay conditions. However, the 24-hour culture was too long for robust cell culture in the absence of serum. Serum was present up to 1 hour before the assay, when cell culture media was exchanged for the standard assay media. The measured OCR was corrected for the relative amounts of viable cells that contributed to the measured OCR, removing the possible interpretation of OCR reductions as being due to cell loss.

The parameter values for ubrogepant were determined by reproducing the cell count-corrected OCR data in MITOsym®, a mechanistic model of mitochondrial function in the *in vitro* setting. MITOsym parameters were then translated to DILIsym parameters using a scaling factor calculated using classic exemplar compounds that perturb different pathways of mitochondrial respiration (e.g., rotenone for ETC inhibition, FCCP for uncoupling).

The concentration of test compound at the site of action (i.e., hepatocytes) has been identified as highly influential in the selection of parameter values. To assess intracellular concentration of ubrogepant, identical HepG2 cultures were established in parallel and cell lysate concentrations of ubrogepant were measured by LC/MS/MS analysis. Intracellular concentrations of ubrogepant were calculated by correcting for cell lysate volume (100 µL) and volume of HepG2 cells in each well, which was calculated by multiplying cell count per well by cellular volume per cell (2.85 pl/cell). One independent study in duplicate was conducted to assess intracellular concentrations of ubrogepant.

### Oxidative stress due to ubrogepant

To assess the potential for ubrogepant to induce oxidative stress, assays to measure formation of reactive oxygen species (ROS) were conducted by Cyprotex. HepG2 cells were cultured in triplicate with ubrogepant for 6 or 24 hours at concentrations ranging from 0.01 – 150 μM, and ROS formation was assayed by DHE fluorescence assay. HepG2 cells have reduced metabolic activity relative to primary human hepatocytes.^3,4^ Three independent studies in triplicate were conducted on HepG2 cells.

Parameter values for ubrogepant-mediated induction of oxidative stress were identified by simulating the experimental data in DILIsym using a DILIsym drug exposure scheme designed to represent *in vitro* conditions.

The concentration of test compound at the site of action (i.e., hepatocytes) has been identified as highly influential in the selection of parameter values. To assess intracellular concentration of ubrogepant, identical HepG2 cultures were established in parallel and cell lysate concentrations of ubrogepant were measured by LC/MS/MS analysis. Intracellular concentrations of ubrogepant were calculated by correcting for cell lysate volume (100 µL) and volume of HepG2 cells in each well, which was calculated by multiplying cell count per well by cellular volume per cell (2.85 pl/cell for HepG2 cells). One independent study in triplicate was conducted to assess intracellular concentrations of ubrogepant.

### Bile acid transporter inhibition by MK-3207

To assess the functional impact of MK-3207 on the human ABC efflux transporters BSEP, MRP3 and MRP4, *in vitro* vesicular transport inhibition assays were conducted by Solvo Biotechnology (Szeged, Hungary) and overseen by DSSI. BSEP transport was assessed in the presence of 0.5-30 μM MK-3207; MRP3 and MRP4 transport was assessed in the presence of 0.11-80 μM MK-3207. The upper limits of these ranges were determined by compound solubility in each assay buffer. NTCP transporter inhibition was assessed in NTCP-expressing HEK293 cells in the presence of 0.03 – 25 μM MK-3207.

IC_50_ values for MK-3207 were calculated for each of the transporters; these values were used as inputs into DILIsym. It was assumed that inhibition type for MK-3207 for MRP3 and MRP4 was mixed with α= 5; this is a typical value for bile acid transporter inhibitors in the experience of the DSS team. However, preliminary simulations suggested that mode of inhibition of BSEP was important in toxicity predictions, so further experiments were undertaken in order to determine the actual K_i_ and mode of inhibition.

In these experiments, taurocholic acid (TCA) transport was assessed at a range of MK-3207 concentrations (0.04 – 30 µM) as well as at a range of TCA media concentrations (0.39 – 50 μM). A K_i_ was then determined by fitting a mixed inhibition model to the inhibition data by nonlinear regression analysis. The fitting process revealed that while the competitive inhibition model appeared to provide a reasonable fit to the inhibition data, the mixed inhibition model could also fit the inhibition data due to mild inhibition by MK-3207. Simulations were therefore performed using the fits to both models; however, the main project conclusions were to be based on the competitive inhibition assumption, which showed the lowest 95% CI interval range from the Solvo study.

### Mitochondria toxicity due to MK-3207

To assess the potential mitochondrial liabilities of MK-3207, cellular respiration assays using a Seahorse XFe96 Flux Analyzer were conducted by Cyprotex (Macclesfield, UK). HepG2 cells were incubated with MK-3207 at concentrations ranging from 0.01 – 75 μM for 1 or 24 hours and tested for an effect on cellular respiration as measured by the OCR. Three independent studies were conducted in duplicate. Importantly, the 1-hour culture was conducted in the absence of serum (i.e., no serum protein binding) as is standard for the Seahorse Analyzer assay conditions. However, the 24-hour culture was too long for robust cell culture in the absence of serum. Serum was present up to 1 hour before the assay, when cell culture media was exchanged for the standard assay media. Intracellular MK-3207 concentrations were also assessed in identical duplicate HepG2 cultures. The measured OCR was corrected for the relative amounts of viable cells that contributed to the measured OCR, removing the possible interpretation of OCR reductions as being due to cell loss; the process for this was the same as that described above for ubrogepant.

Mitochondrial toxicity parameter values for MK-3207 were determined by reproducing the cell count-corrected OCR response to MK-3207 dosing in both MITOsym and DILIsym. The ETC inhibition coefficient from the MITOsym parameters was then translated to a DILIsym parameter using a scaling factor calculated using classic exemplar compounds that perturb different pathways of mitochondrial respiration (e.g., rotenone for ETC inhibition, FCCP for uncoupling), similar to ubrogepant. The maximum inhibition potency from the DILIsym fit was combined with this value in the final parameter set.

### Oxidative stress due to MK-3207

To assess the potential for MK-3207 to induce oxidative stress, assays to measure formation of ROS were conducted by Cyprotex. HepG2 cells were cultured in triplicate with MK-3207 for 6 or 24 hours at concentrations ranging from 0.01 – 150 μM and ROS formation was assayed by DHE fluorescence assay. HepG2 cells have reduced metabolic activity relative to primary human hepatocytes.^3,4^ Three independent studies in triplicate were conducted.

Parameter values for MK-3207-mediated induction of oxidative stress were identified by simulating the experimental data in DILIsym using a DILIsym drug exposure scheme meant to represent *in vitro* conditions. Intracellular compound concentrations were also assayed in identical cell cultures; the experimental method was the same as that described above for ubrogepant.

# **Assay Results**

### Bile acid transporter Inhibition by telcagepant

In vesicular and cellular transport inhibition assays, telcagepant inhibited BSEP-, MRP3-, MRP4‑, or NTCP-mediated probe substrate accumulation dose dependently (Figure 1); the maximum inhibition at the highest telcagepant concentration tested (20 µM; solubility limit) was < 50% for MRP3 and MRP4.

NTCP was inhibited > 50% with an estimated IC_50_ of 19.4 µM. Telcagepant also showed BSEP inhibition > 50% with an estimated IC_50_ of 10.2 µM . As a result, additional studies were conducted with multiple substrate concentrations and multiple inhibitor concentrations to determine the K_i_ and inhibition type of telcagepant for BSEP (Figure 2). Both K_m_ and V_max_ were altered with increasing inhibitor concentration, suggesting that telcagepant is likely to be a mixed BSEP inhibitor; K_m_ tended to increase with increasing inhibitor concentration, whereas V_max_ increased at low inhibitor concentrations, but then decreased (Table 1).

### Mitochondria toxicity due to telcagepant

In the mitochondrial respiration assay, 1-hour incubation with telcagepant was found to inhibit the HepG2 OCR in a dose-dependent manner ([Figure](#Fig_7) 3). A similar OCR response was measured following 24-hour incubation with telcagepant ([Figure](#Fig_8) 4), suggesting that telcagepant acts as a mitochondrial ETC inhibitor. Because the pattern of response was similar at 1 and 24 hours, and interpretation of the 24-hour data was complicated by cytotoxicity, the 1-hour data were prioritized for use to support the subsequent simulations.

### Oxidative stress due to telcagepant

In the oxidative stress assay, there was evidence for modest induction of oxidative stress in HepG2 cells following 24-hour incubation with telcagepant (Figure 5).

### Bile acid transporter inhibition by ubrogepant

In vesicular and cellular transport inhibition assays, ubrogepant inhibited BSEP- and MRP3-mediated probe substrate accumulation dose dependently (Figure 6); the maximum inhibition at the highest ubrogepant concentration tested (70 µM; solubility limit) was < 50% for MRP3, MRP4, and NTCP, though MRP3 inhibition was significant enough for the DSSI team to calculate an IC_50_ value.

Additional studies were conducted with multiple substrate concentrations and multiple inhibitor concentrations to determine the K_i_ and inhibition type of ubrogepant for BSEP (Figure 7). Both K_m_ and V_max_ were altered with increasing inhibitor concentration, suggesting that ubrogepant is likely to be a mixed BSEP inhibitor; K_m_ tended to increase and V_max_ tended to decrease with increasing inhibitor concentration (Table 2).

### Mitochondria toxicity due to ubrogepant

In the mitochondrial respiration assay, ubrogepant was found to inhibit the HepG2 OCR in a dose-dependent manner after a 24-hour incubation (Figure 8) but not after a 1-hour incubation (Figure 9), suggesting that ubrogepant acts as a mitochondrial ETC inhibitor. Because the effect was more pronounced after 24 hours than after 1 hour, the 24-hour data were used to calculate the DILIsym ETC inhibition parameter for ubrogepant.

### Oxidative stress due to ubrogepant

In the oxidative stress assay, there was evidence for modest induction of oxidative stress in HepG2 cells following 24-hour incubation with ubrogepant (Figure 10). This response was not evident following the 6-hour incubation period (Figure 11). Given the reduced metabolic activity in HepG2 cells, the detection of ROS in ubrogepant-treated HepG2 cells, but not HepaRG spheroids, suggests that the parent compound is responsible for the ROS production.

### Bile acid transporter inhibition by MK-3207

In vesicular transport inhibition assays, MK-3207 inhibited BSEP- and MRP3-mediated substrate transport in vesicles. No inhibition was observed for MRP4 or NTCP (Figure 12).

Further studies were conducted with BSEP vesicles in order to determine the K_i_ value and mode of inhibition for MK-3207 (Figure 13). Increasing the concentration of MK-3207 increased the apparent K_m_; however, it was unclear if V_max_ was similarly altered. The inhibition data were fit to both competitive and mixed inhibition models; the competitive model fit marginally better but both models were found to fit the data well. The K_m_ for bile acid transport was clearly increased by MK-3207, but the effect on the V_max_ value was unclear (Table 3).

### Mitochondria toxicity due to MK-3207

MK-3207 was found to mildly inhibit cellular respiration after both a 1-hour (Figure 8) and a 24-hour (Figure 9) incubation in HepG2 cells. The observed OCR inhibition was minor but consistent across a broad concentration range. The data are consistent with a saturable inhibition of the mitochondrial ETC.

### Oxidative stress due to MK-3207

In the oxidative stress assay, there was evidence for modest induction of oxidative stress in HepG2 cells following 24-hour incubation with MK-3207 (Figure 10). This response was not evident following the 6-hour incubation period (Figure 11).

# **Translating In vitro Assay Data to DILIsym^®^ Parameter Values**

For the simulation of CGRP inhibitors in DILIsym®, the *in vitro* assay data were translated to DILIsym® parameter values. The method of translation varied by the type of data available and the corresponding mechanism in DILIsym®. The following sections detail this translation for bile acid transporter inhibition, mitochondrial toxicity, and oxidative stress.

### Bile acid transporter inhibition by telcagepant

The mixed inhibition model best described the telcagepant mediated BSEP inhibition with a K_i_ of 7.9 µM and alpha of 4.6 (Figure 14). The estimated IC_50_ value of telcagepant for NTCP was 19.4 µM. The maximum inhibition at the highest telcagepant concentration tested (20 µM; solubility limit) was < 50% for MRP3 and MRP4. Therefore, the lowest possible IC_50_ was estimated by curve fitting, assuming the maximum inhibition at higher concentrations is 100%. The estimated lowest possible IC_50_ values of telcagepant for MRP3 and MRP4 were 16.6 and 16.9 µM, respectively (Figure 15). Because basolateral efflux of bile acids is represented as a single transport process in DILIsym®, the IC_50_ value of telcagepant for MRP3 was used as the inhibition constant for bile acid basolateral efflux in the simulations; this is a conservative approach, since the IC_50_ for MRP3 was slightly lower than the IC_50_ for MRP4. Because K_i_ studies were not performed for NTCP, MRP3, or MRP4, mixed inhibition with an alpha of 4.6 was assumed for these transporters, similar to BSEP.

### Mitochondria toxicity due to telcagepant

To define the DILIsym® parameter values for telcagepant-mediated mitochondrial toxicity, the 1 hour *in vitro* data were simulated within MITOsym® (Figure 16). The intracellular concentration of telcagepant was measured by LC/MS/MS analysis; the median measured cell-to-media ratio (56.1) was used to estimate intracellular concentrations at each dose. Reproduction of telcagepant-mediated inhibition of mitochondrial respiration in MITOsym® defined telcagepant as an electron transport chain (ETC) inhibitor, with an ETC inhibition constant of 0.5 mM. This MITOsym® parameter was translated to a DILIsym® ETC inhibition constant of 1.74 x 10^-5^ mol/mL, using a scaling factor of 34.7 derived from the relative activity of rotenone in HepG2 cells and *in vivo*.

### Oxidative stress due to telcagepant

To define the DILIsym® parameter values for telcagepant-mediated oxidative stress, the 24-hour *in vitro* data were simulated within DILIsym® (Figure 17). The intracellular concentration of telcagepant was measured by LC/MS/MS analysis; the median measured cell-to-media ratio (56.1) was used to estimate intracellular concentrations at each dose. Reproduction of telcagepant-mediated induction of oxidative stress defined the relationship between liver compound concentration and ROS formation with the RNS/ROS production rate constant of 20,000 mL/mol/hr.

### Bile acid transporter inhibition by ubrogepant

The mixed inhibition model best described the ubrogepant-mediated BSEP inhibition with a K_i_ of 38.1 µM and alpha of 8.39 (Figure 7). The maximum inhibition at the highest ubrogepant concentration tested (70 µM; solubility limit) was < 50% for MRP3 and MRP4. Therefore, the DILIsym team estimated the lowest possible IC_50_ by curve fitting, assuming the maximum inhibition at higher concentrations is 100%. The estimated lowest possible ubrogepant IC_50_ value for MRP3 was 85.9 µM (Figure 18). Because basolateral efflux of bile acids is represented as a single transport process in DILIsym, the ubrogepant IC_50_ value for MRP3 was used as the inhibition constant for bile acid basolateral efflux in the simulations; this is a conservative approach, since ubrogepant did not inhibit MRP4. Because K_i_ studies were not performed for MRP3, mixed inhibition with an alpha of 5 was assumed for these transporters.

### Mitochondria toxicity due to ubrogepant

To define the DILIsym parameter values for ubrogepant-mediated mitochondrial ETC inhibition, the 24-hour *in vitro* data were simulated within MITOsym (Figure 19). The intracellular concentration of ubrogepant was measured by LC/MS/MS analysis and the cell count was measured as part of this analysis; the data were corrected for both intracellular concentration and cell count. Reproduction of ubrogepant-mediated inhibition of mitochondrial respiration in MITOsym defined ubrogepant as an ETC inhibitor, with an ETC inhibition constant of 0.0136 mM. This MITOsym parameter was translated to a DILIsym ETC inhibition constant of 472 μM, using a scaling factor of 34.7 derived from the relative activity of rotenone in HepG2 cells and *in vivo*.

### Oxidative stress due to ubrogepant

To define the DILIsym parameter values for ubrogepant-mediated oxidative stress, the 24-hour *in vitro* data were simulated within DILIsym (Figure 20). The intracellular concentration of ubrogepant was measured by LC/MS/MS analysis; the data were corrected for these values. Reproduction of ubrogepant-mediated induction of oxidative stress defined the relationship between liver compound concentration and ROS formation with the RNS/ROS production rate constant of 1.6 x 10^-4^ mL/nmol/hr.

### Bile acid transporter inhibition by MK-3207

BSEP K_i_ values were calculated for MK-3207 based on the fit of the competitive inhibition model to the vesicle assay data (Figure 13). As previously mentioned, the competitive model is presumed to be the most likely mode of inhibition; however, because the measured inhibition of bile acid transport by MK-3207 plausibly resembles both competitive and mixed inhibition, K_i_ values were calculated and simulations were performed for both of the potential modes of inhibition. The K_i_ value for competitive inhibition was 7.62 μM.

For MRP3, the calculated IC_50_ of 49.9 μM was used as the basolateral transporter inhibition constant; mixed inhibition with alpha = 5 was assumed as the basolateral transporter inhibition mode.

### Mitochondria toxicity due to MK-3207

The MK-3207 1-hour and 24-hour HepG2 OCR dose response data were simulated with both MITOsym and DILIsym (Figure 21) in order to determine ETC inhibition input parameters. The intracellular concentration of MK-3207 was measured by LC/MS/MS analysis; the data were corrected for these values as well as for cell count. The inhibition constant from MITOsym was translated into DILIsym and combined with the maximal inhibition parameter derived from the DILIsym fit. The ETC inhibition coefficient was calculated to be 0.347 μM with a maximal inhibition value of 0.35.

### Oxidative stress due to MK-3207

To define the DILIsym parameter values for MK-3207 mediated oxidative stress, the 24-hour *in vitro* data were simulated within DILIsym (Figure 22). The intracellular concentration of MK-3207 was measured by LC/MS/MS analysis; the data were corrected for these values. Reproduction of MK-3207 mediated induction of oxidative stress defined the relationship between liver compound concentration and ROS formation with the RNS/ROS production rate constant of 2.2 x 10^-4^ mL/nmol/hr.

**References**

1. Woodhead JL, Yang K, Brouwer KLR, Siler SQ, Stahl SH, Ambroso JL, et al. Mechanistic modeling reveals the critical knowledge gaps in bile acid-mediated DILI. *CPT Pharmacometrics Syst Pharmacol*. 2014;3:e123. doi: 10.1038/psp.2014.21.

2. Yang K, Woodhead JL, Watkins PB, Howell BA, Brouwer KL. Systems pharmacology modeling predicts delayed presentation and species differences in bile acid-mediated troglitazone hepatotoxicity. *Clin Pharmacol Ther*. 2014;28:589–598.

3. Woodhead JL, Watkins PB, Howell BA, Siler SQ, Shoda LKM. The role of quantitative systems pharmacology modeling in the prediction and explanation of idiosyncratic drug-induced liver injury. *Drug Metab Pharmacokinet*. 2017;32(1):40–45.

4. Yang Y, Nadanaciva S, Will Y, Woodhead JL, Howell BA, Watkins PB, et al. MITOsym®: A mechanistic, mathematical model of hepatocellular respiration and bioenergetics. *Pharm Res*. 2015;32:1975–1992.

# **Supplementary Figures**

Figure 1. Inhibition of bile acid transporters by telcagepant (MK-0974). (a) Inhibition of BSEP-mediated taurocholate (TC) transport by telcagepant in the vesicular transport inhibition assay, (b) inhibition of MRP3-mediated estradiol-17-β-glucuronide (E217βG) transport by telcagepant in the vesicular transport inhibition assay, (c) inhibition of MRP4-mediated dehydroepiandrosterone sulfate (DHEAS) transport by telcagepant in the vesicular transport inhibition assay, and (d) inhibition of NTCP-mediated taurocholate transport by telcagepant in the uptake transporter inhibition assay.


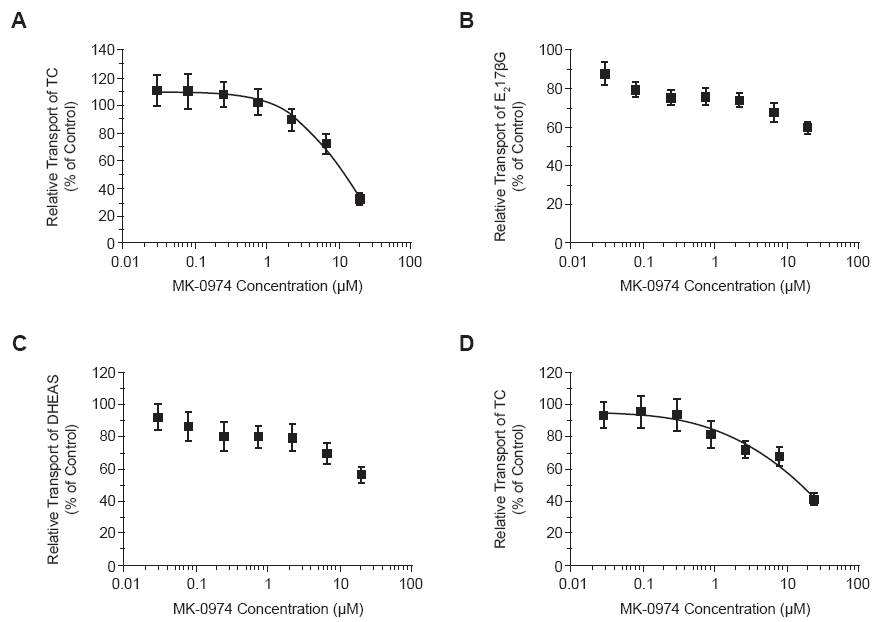


Figure 2. Inhibition of BSEP-mediated taurocholate (TC) transport by telcagepant in the vesicular transport inhibition assay. Each data point (symbols) represent mean ± SD of triplicate measurements from n=1 independent study.


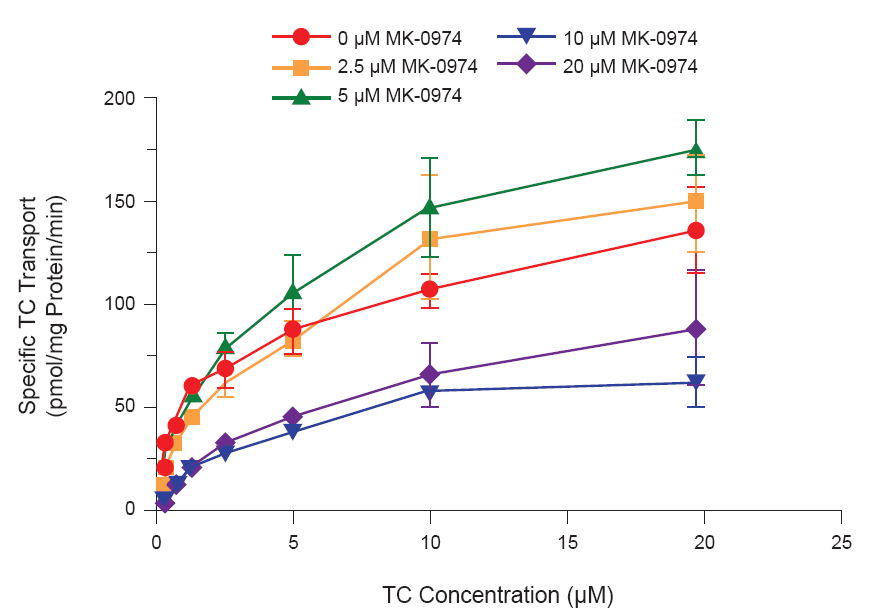


Figure 3. Inhibition of oxygen consumption rate (OCR) by telcagepant following 1-hour incubation with HepG2 cells in the cellular respiration assay, conducted in three independent experiments (a-c). Filled blue diamonds are mean data points for each concentration, plus or minus standard deviation. Open blue circles are data points excluded from the plot due to data plateau or other reasons. Green dashed lines delineate significant cut-off from vehicle control.


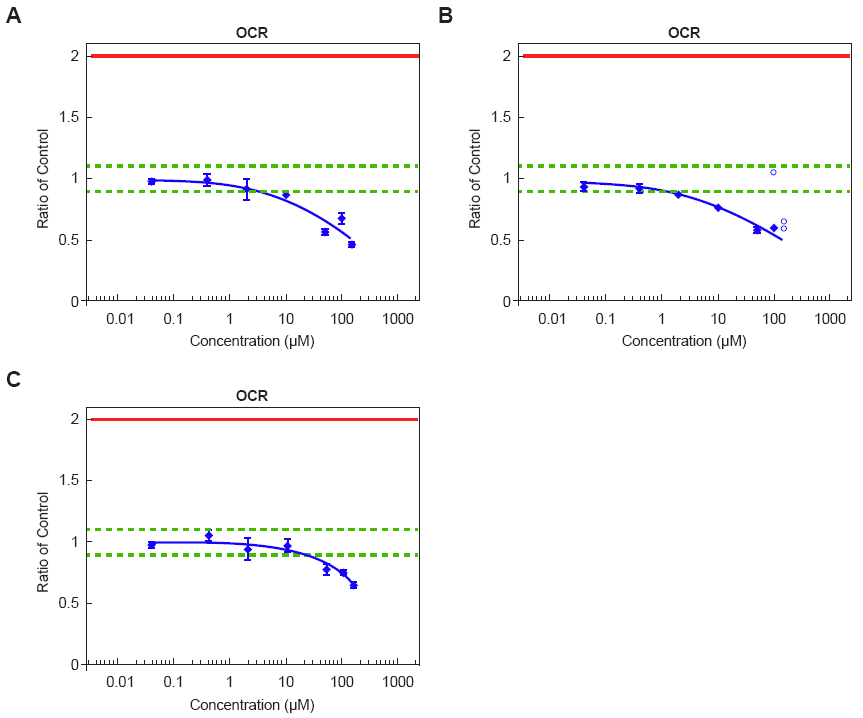


Figure 4. Inhibition of oxygen consumption rate (OCR) by telcagepant following 24-hour incubation with HepG2 cells in the cellular respiration assay, conducted in three independent experiments (a-c). Filled blue diamonds are mean data points for each concentration, plus or minus standard deviation. Open blue circles are data points excluded from the plot due to data plateau or other reasons. Green dashed lines delineate significant cut-off from vehicle control.


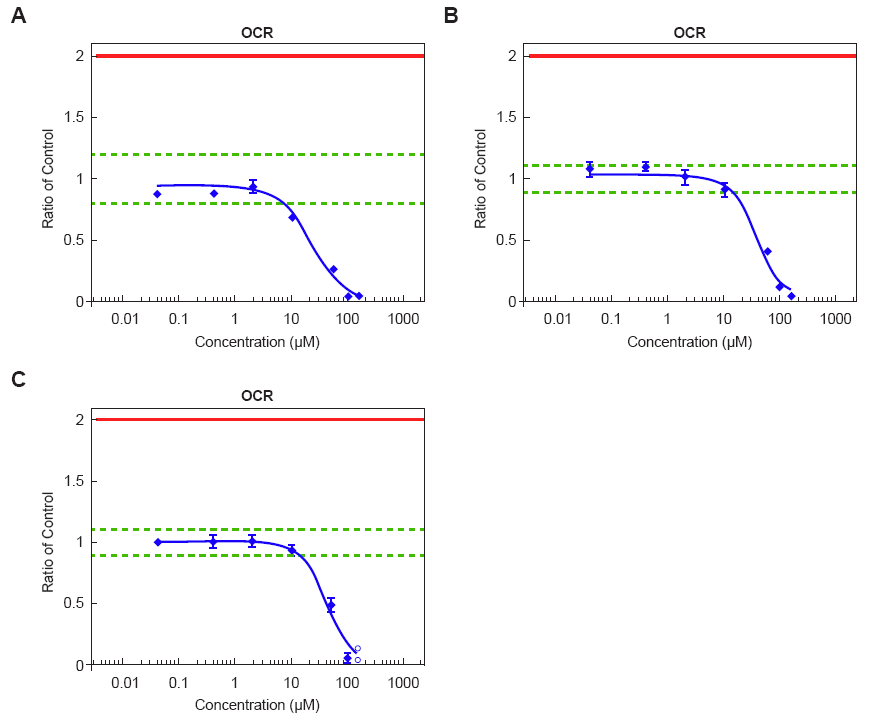


Figure 5. Formation of reactive oxygen species (ROS) following 24-hour incubation of telcagepant with HepG2 cells in the oxidative stress assay, conducted in three independent experiments (a-c). Filled blue diamonds are mean data points for each concentration, plus or minus standard deviation. Open blue circles are data points excluded from the plot due to data plateau or other reasons. Green dashed lines delineate significant cut-off from vehicle control .


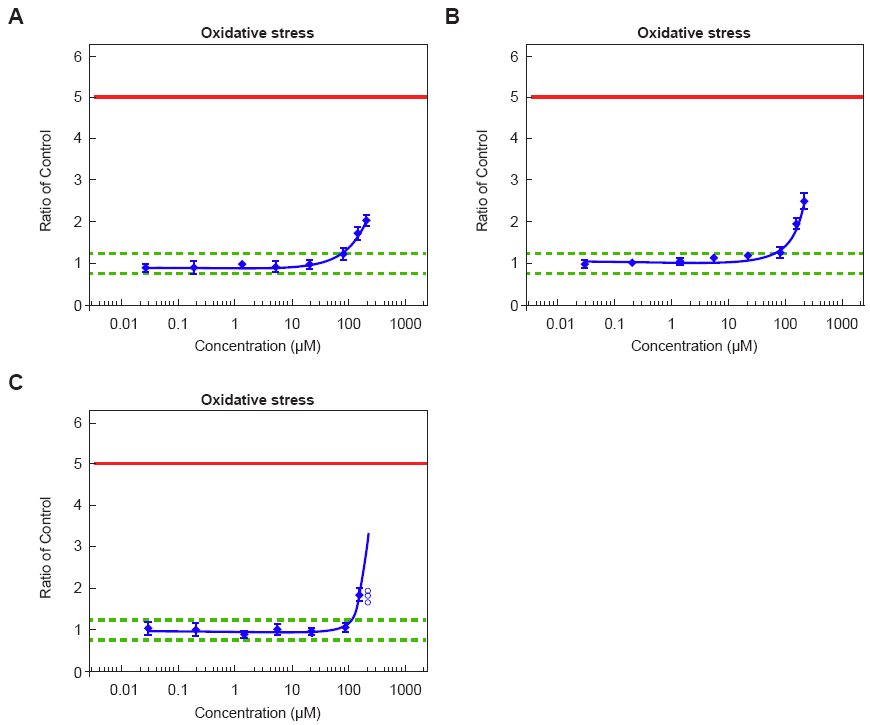


Figure 6. Inhibition of bile acid transporters by ubrogepant. (a) Inhibition of BSEP-mediated taurocholate transport by ubrogepant in the vesicular transport inhibition assay, (b) inhibition of MRP3-mediated estradiol-17-β-glucuronide (E217βG) transport by ubrogepant in the vesicular transport inhibition assay, (c) inhibition of MRP4-mediated dehydroepiandrosterone sulfate (DHEAS) transport by ubrogepant in the vesicular transport inhibition assay, and (d) inhibition of NTCP-mediated taurocholate transport by ubrogepant in the uptake transporter inhibition assay.


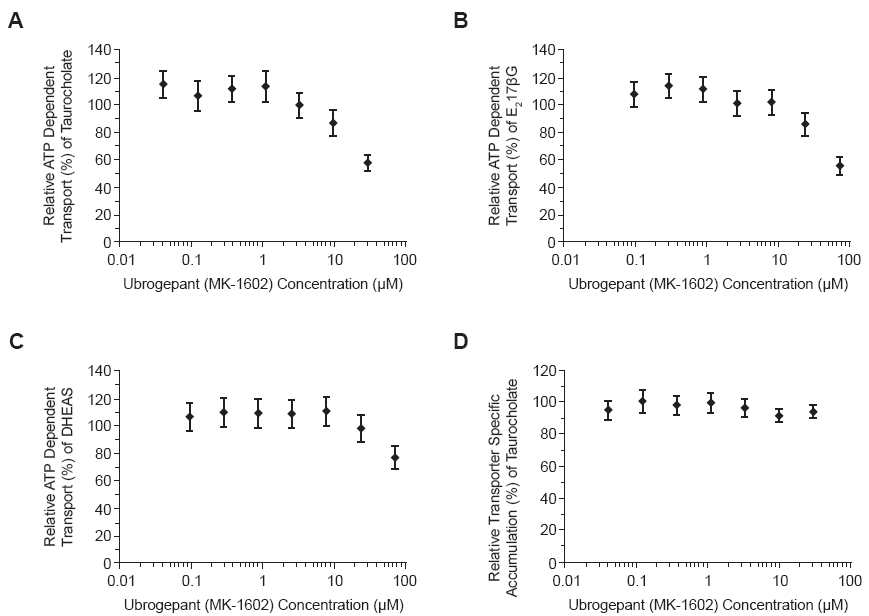


Figure 7. Inhibition of BSEP-mediated taurocholate (TC) transport by ubrogepant in the vesicular transport inhibition assay. Each data point (symbols) represents mean ± SD of triplicate measurements from n=1 independent study.


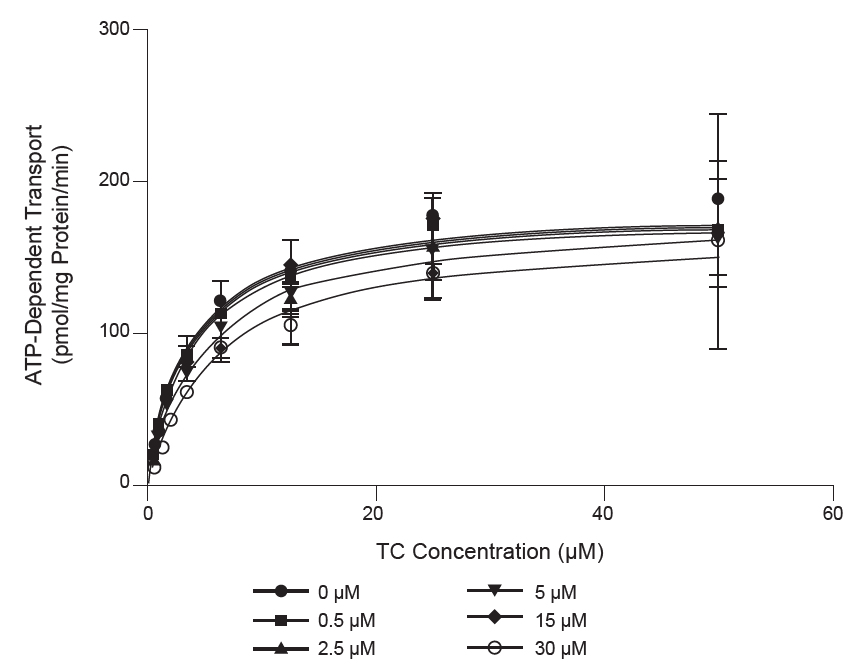


Figure 8. Inhibition of oxygen consumption rate (OCR) by ubrogepant (MK-1602; black circles) and MK-3207 (blue diamonds) following 24-hour incubation with HepG2 cells in the cellular respiration assay, conducted in three independent experiments. The data have been corrected for the intracellular concentration of each compound and for the cell count in each assay.


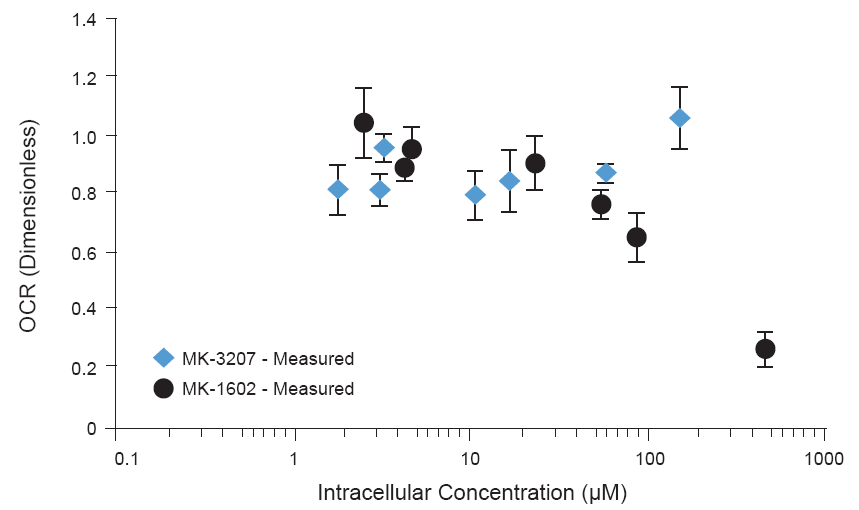


Figure 9. Inhibition of oxygen consumption rate (OCR) by ubrogepant (MK-1602; black circles) and MK-3207 (blue diamonds) following 1-hour incubation with HepG2 cells in the cellular respiration assay, conducted in three independent experiments. The data have been corrected for the intracellular concentration of each compound and for the cell count in each assay.


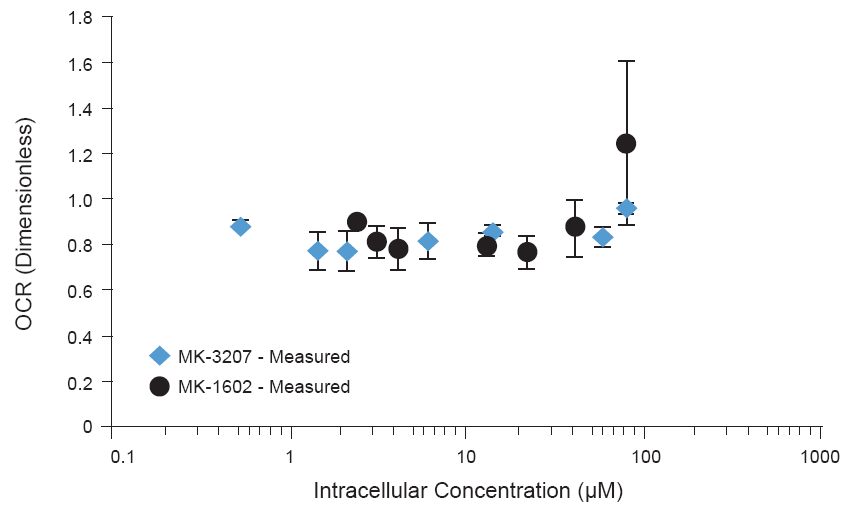


Figure 10. Formation of reactive oxygen species (ROS) following 24-hour incubation of ubrogepant (MK-1602; black circles) and MK-3207 (blue diamonds) with HepG2 cells in the oxidative stress assay, conducted in three independent experiments. The data have been corrected for the intracellular concentration of each compound.


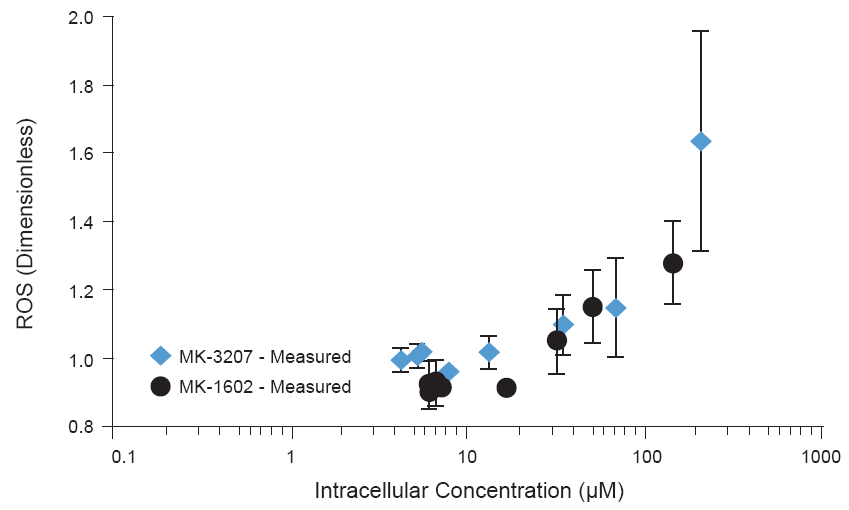


Figure 11. Formation of reactive oxygen species (ROS) following 6-hour incubation of ubrogepant (MK-1602; black circles) and MK-3207 (blue diamonds) with HepG2 cells in the oxidative stress assay, conducted in three independent experiments. The data have been corrected for the intracellular concentration of each compound.


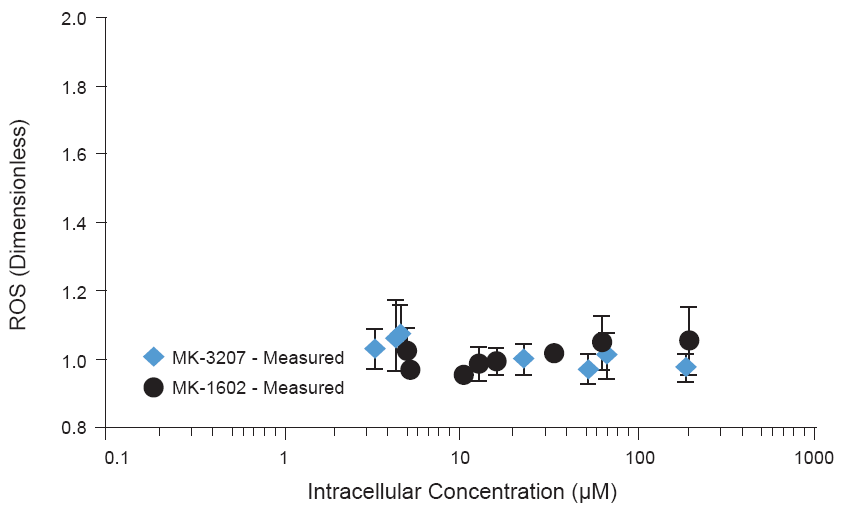


Figure 12. Inhibition of bile acid transporters by MK-3207. (a) Inhibition of BSEP-mediated taurocholate (TC) transport by MK-3207 in the vesicular transport inhibition assay, (b) inhibition of MRP3-mediated estradiol-17-β-glucuronide (E217βG) transport by MK-3207 in the vesicular transport inhibition assay, (c) inhibition of MRP4-mediated dehydroepiandrosterone sulfate (DHEAS) transport by MK-3207 in the vesicular transport inhibition assay, and (d) inhibition of NTCP-mediated taurocholate transport by MK-3207 in the uptake transporter inhibition assay.


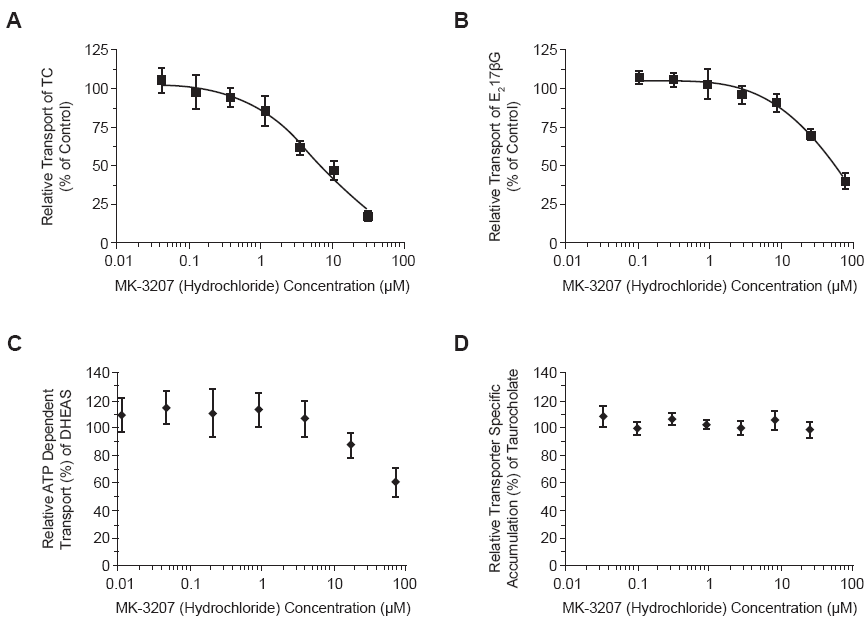


Figure 13. Inhibition of BSEP-mediated taurocholate (TC) transport by MK-3207 in the vesicular transport inhibition (K_i_) assay using the competitive model (a) and the mixed model (b). Each data point (symbols) represents mean ± SD of triplicate measurements from n=1 independent study.


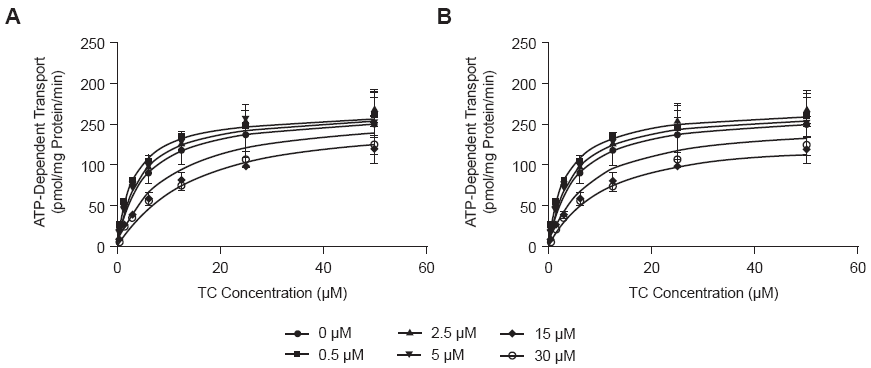


Figure 14. Analysis of K_i_ and inhibition type of telcagepant (MK-0974) for BSEP inhibition by nonlinear regression analysis. Each data point (symbols) represent mean ± SD of triplicate measurements from n=1 independent study. The curves represent the best fit model (mixed inhibition model) using nonlinear regression analysis.


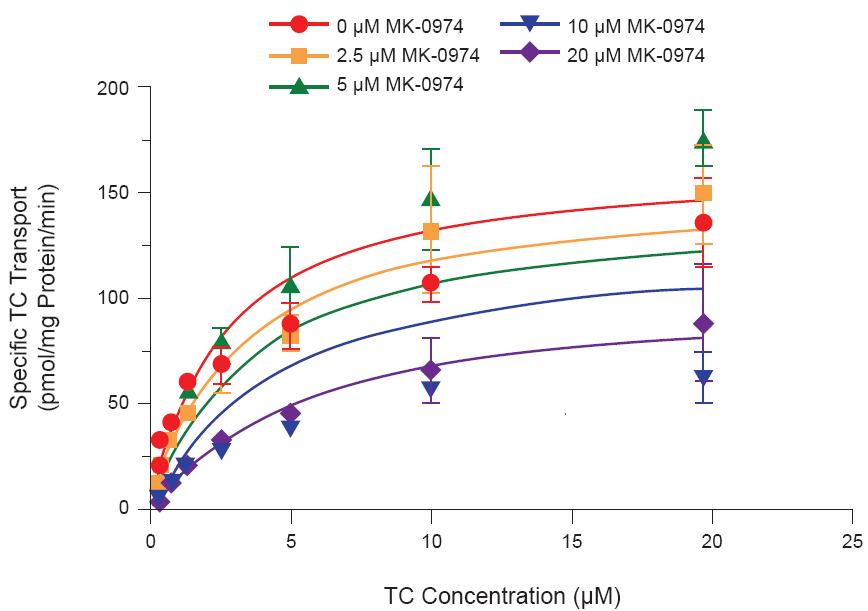


Figure 15. Analysis of IC_50_ of telcagepant for inhibition of MRP3 (a) and MRP4 (b).


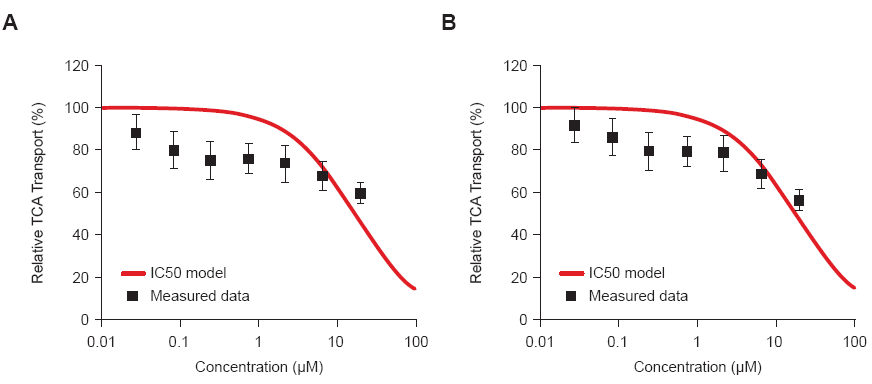


Figure 16. Comparison of simulation results and *in vitro* assay data to identify DILIsym® parameter values that reproduce the concentration-dependent relationship between telcagepant (MK-0974) and mitochondrial toxicity.


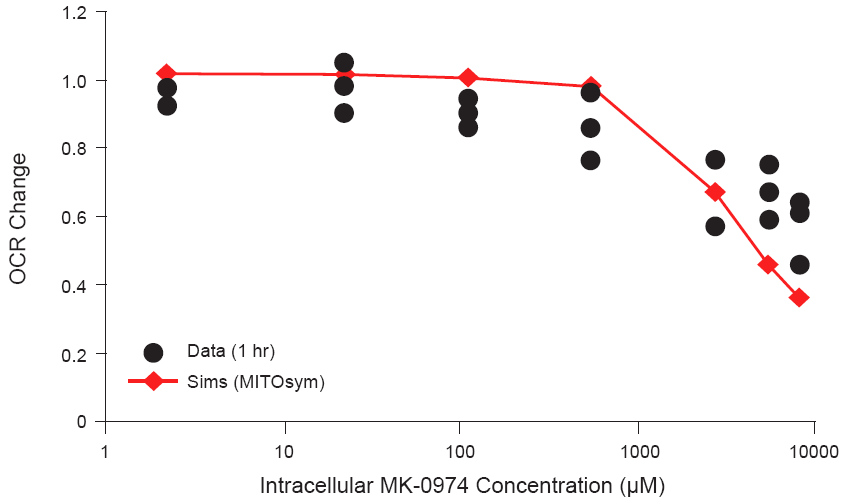


Figure 17. Comparison of simulation results and *in vitro* assay data to identify DILIsym® parameter values that reproduce the concentration-dependent relationship between telcagepant (MK-0974) and oxidative stress.


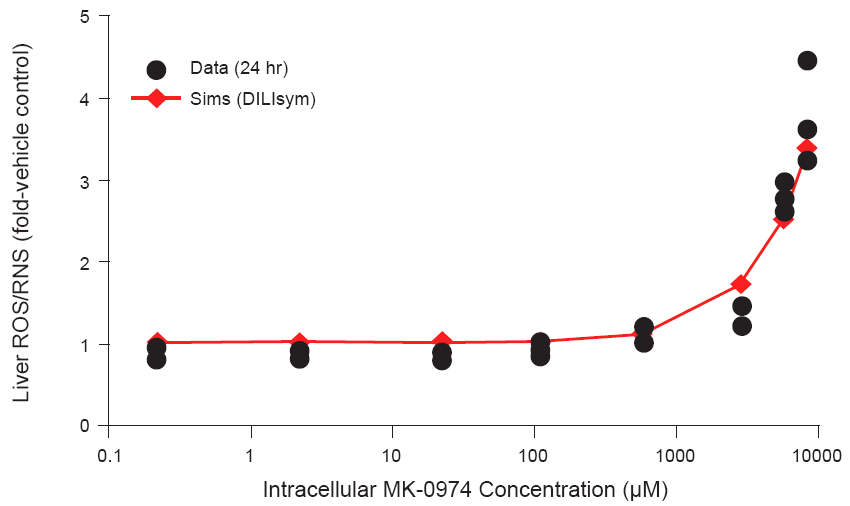


Figure 18. Analysis of IC_50_ of ubrogepant (MK-1602) for inhibition of MRP3.


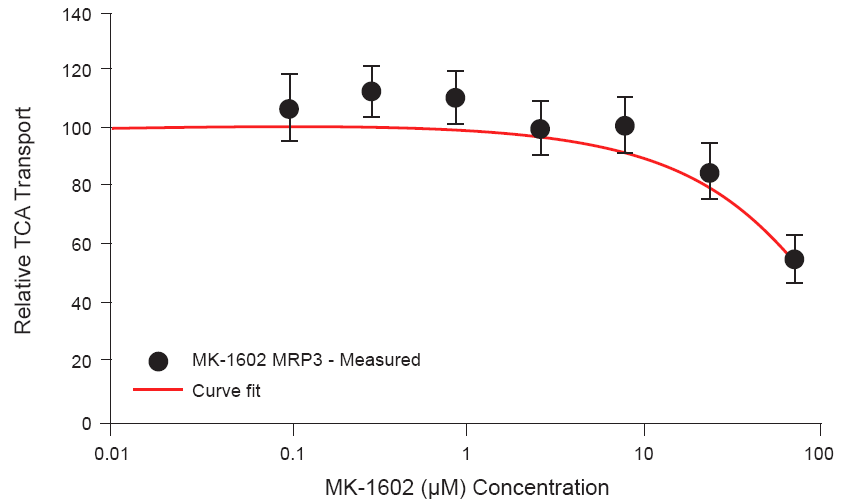


Figure 19. Comparison of simulation results and *in vitro* assay data to identify DILIsym parameter values that reproduce the concentration-dependent relationship between ubrogepant (MK-1602) and mitochondrial OCR reduction.


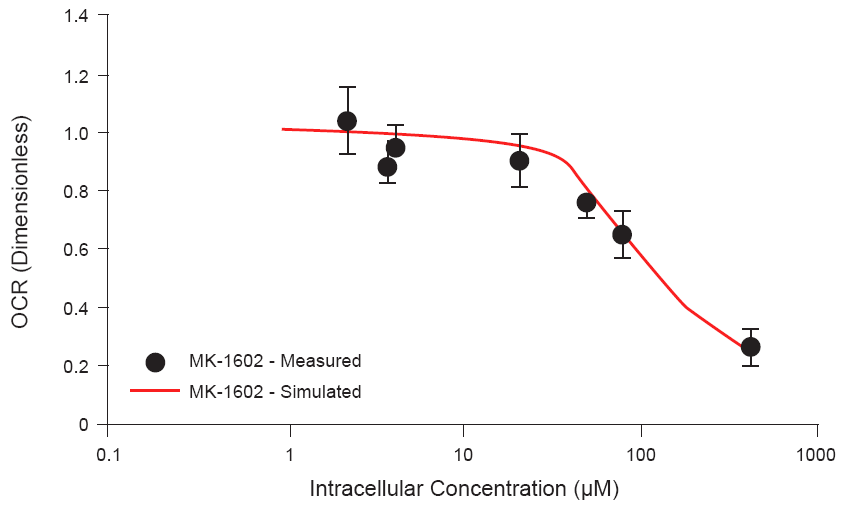


Figure 20. Comparison of simulation results and *in vitro* assay data to identify DILIsym parameter values that reproduce the concentration-dependent relationship between ubrogepant (MK-1602) and oxidative stress.


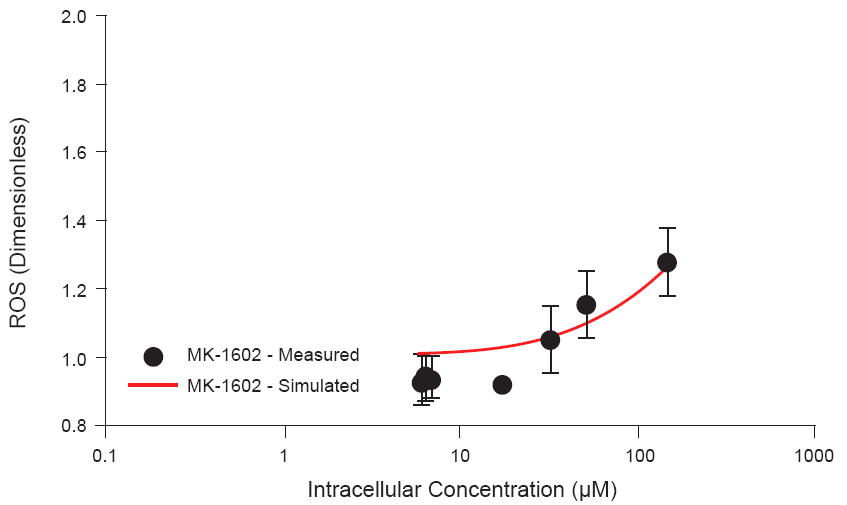


Figure 21. Comparison of MITOsym and DILIsym simulation results and *in vitro* assay data after 1-hour (a) and 24-hour (b) incubations used to identify DILIsym parameter values that reproduce the concentration-dependent relationship between MK-3207 and mitochondrial toxicity. The MITOsym fit that produced the ETC inhibition 3 coefficient is represented by the solid red line; the DILIsym fit that produced the maximal inhibition value is represented by the dashed red line.


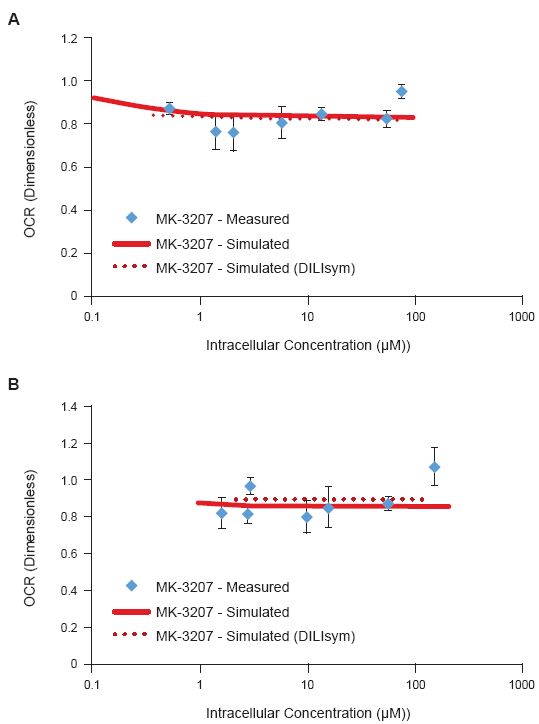


Figure 22. Comparison of simulation results and *in vitro* assay data to identify DILIsym parameter values that reproduce the concentration-dependent relationship between MK-3207 and oxidative stress after the 24-hour incubation.


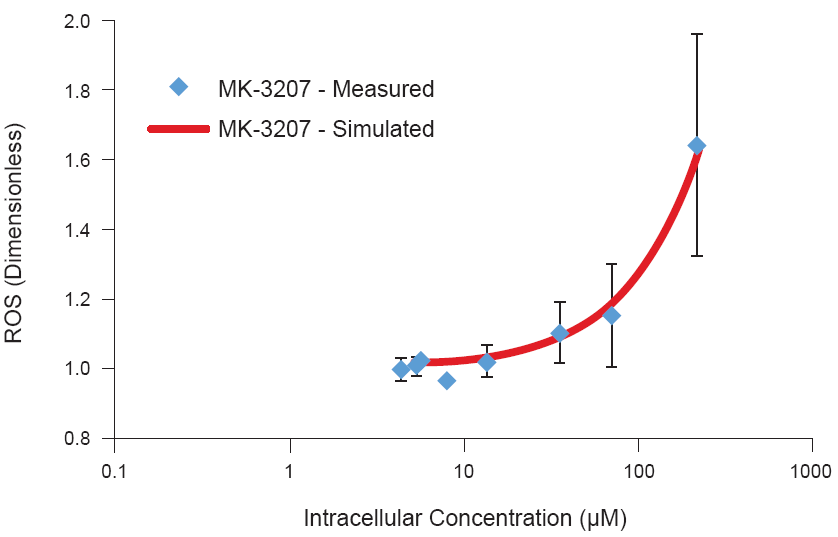


# **Supplementary Tables**

Table 1. Michaelis-Menten kinetic parameters of taurocholate in the BSEP vesicle assay in the presence of various concentrations of telcagepant.

| Telcagepant concentration (µM) | 0 | 2.5 | 5 | 10 | 20 |
| --- | --- | --- | --- | --- | --- |
| V_max_ (pmol/mg protein/min) | 135.8 | 186.3 | 208.1 | 77.52 | 119.7 |
| K_m_ (µM) | 1.786 | 4.388 | 3.756 | 3.844 | 6.946 |

Table 2. Michaelis-Menten kinetic parameters of taurocholate in the BSEP vesicle assay in the presence of various concentrations of ubrogepant.

| Ubrogepant concentration (µM) | 0 | 0.5 | 2.5 | 5 | 15 | 30 |
| --- | --- | --- | --- | --- | --- | --- |
| V_max_ (pmol/mg protein/min) | 200.4 | 181.3 | 175.6 | 171.3 | 171.9 | 172.3 |
| K_m_ (µM) | 3.849 | 3.233 | 3.68 | 3.773 | 4.376 | 5.713 |

Table 3. Michaelis-Menten kinetic parameters of taurocholate in the BSEP vesicle assay in the presence of various concentrations of MK-3207.

| MK-3207 concentration (µM) | 0 | 0.5 | 2.5 | 5 | 15 | 30 |
| --- | --- | --- | --- | --- | --- | --- |
| V_max_ (pmol/mg protein/min) | 159.1 | 171.9 | 183.1 | 174.9 | 132.5 | 151.7 |
| K_m_ (µM) | 3.367 | 3.525 | 4.789 | 4.821 | 7.417 | 11.1 |
